# MitoSort: Robust Demultiplexing of Pooled Single-cell Genomic Data Using Endogenous Mitochondrial Variants

**DOI:** 10.1093/gpbjnl/qzae073

**Published:** 2024-10-15

**Authors:** Zhongjie Tang, Weixing Zhang, Peiyu Shi, Sijun Li, Xinhui Li, Yueming Li, Yicong Xu, Yaqing Shu, Zheng Hu, Jin Xu

**Affiliations:** State Key Laboratory of Biocontrol, School of Life Sciences, Sun Yat-sen University, Guangzhou 510275, China; State Key Laboratory of Biocontrol, School of Life Sciences, Sun Yat-sen University, Guangzhou 510275, China; State Key Laboratory of Biocontrol, School of Life Sciences, Sun Yat-sen University, Guangzhou 510275, China; State Key Laboratory of Biocontrol, School of Life Sciences, Sun Yat-sen University, Guangzhou 510275, China; State Key Laboratory of Biocontrol, School of Life Sciences, Sun Yat-sen University, Guangzhou 510275, China; State Key Laboratory of Biocontrol, School of Life Sciences, Sun Yat-sen University, Guangzhou 510275, China; State Key Laboratory of Biocontrol, School of Life Sciences, Sun Yat-sen University, Guangzhou 510275, China; Department of Neurology, The Third Affiliated Hospital of Sun Yat-sen University, Guangzhou 510630, China; CAS Key Laboratory of Quantitative Engineering Biology, Shenzhen Institute of Synthetic Biology, Shenzhen Institute of Advanced Technology, Chinese Academy of Sciences, Shenzhen 518055, China; State Key Laboratory of Biocontrol, School of Life Sciences, Sun Yat-sen University, Guangzhou 510275, China

**Keywords:** Single-cell genomics, Demultiplexing, Doublet detection, Mitochondrial genome variant, MitoSort

## Abstract

Multiplexing across donors has emerged as a popular strategy to increase throughput, reduce costs, overcome technical batch effects, and improve doublet detection in single-cell genomic studies. To eliminate additional experimental steps, endogenous nuclear genome variants are used for demultiplexing pooled single-cell RNA sequencing (scRNA-seq) data by several computational tools. However, these tools have limitations when applied to single-cell sequencing methods that do not cover nuclear genomic regions well, such as single-cell assay for transposase-accessible chromatin with sequencing (scATAC-seq). Here, we demonstrate that mitochondrial germline variants are an alternative, robust, and computationally efficient endogenous barcode for sample demultiplexing. We propose MitoSort, a tool that uses mitochondrial germline variants to assign cells to their donor origins and identify cross-genotype doublets in single-cell genomic datasets. We evaluate its performance by using *in silico* pooled mitochondrial scATAC-seq (mtscATAC-seq) libraries and experimentally multiplexed data with cell hashtags. MitoSort achieves high accuracy and efficiency in genotype clustering and doublet detection for mtscATAC-seq data, addressing the limitations of current computational techniques tailored for scRNA-seq data. Moreover, MitoSort exhibits versatility, and can be applied to various single-cell sequencing approaches beyond mtscATAC-seq provided that the mitochondrial variants are reliably detected. Furthermore, we demonstrate the application of MitoSort in a case study where B cells from eight donors were pooled and assayed by single-cell multi-omics sequencing. Altogether, our results demonstrate the accuracy and efficiency of MitoSort, which enables reliable sample demultiplexing in various single-cell genomic applications. MitoSort is available at https://github.com/tangzhj/MitoSort.

## Introduction

Single-cell sequencing techniques have revolutionized the field of genomics by providing an unprecedented level of resolution to study cellular heterogeneity. They offer new insights into the complexity of biological systems and disease processes [1–4]. However, the high costs of single-cell techniques limit their applications in large cohort studies [5]. To meet the growing demands for profiling large-scale samples, multiplexing samples has become a popular strategy. It not only reduces costs, but also overcomes technical batch effects and improves doublet detection [6–11]. To avoid additional experimental processing steps, several computational tools have been proposed to harness nuclear genome variants for demultiplexing cells from different individuals and identifying cross-genotype doublets in single-cell sequencing data, especially for single-cell RNA sequencing (scRNA-seq) data [12–16]. However, this process typically requires significant computational resources due to the large size of the human genome. Furthermore, the performance of these methods may be suboptimal when applied to single-cell sequencing approaches targeting nuclear genome DNA, such as single-cell assay for transposase-accessible chromatin using sequencing (scATAC-seq) [17], as their efficacy relies on the read coverage of the genome variants.

In contrast to the nuclear genome variants, mitochondrial genome variants offer a computationally efficient and robust option for demultiplexing, given their hundreds of copies per cell and the small size of the mitochondrial genome [18,19]. Moreover, mitochondrial genome variants can be efficiently captured at single-cell resolution using multiple single-cell sequencing techniques, such as mitochondrial scATAC-seq (mtscATAC-seq) [20], ATAC with select antigen profiling by sequencing (ASAP-seq) [21], DOGMA-seq [21], and full-length scRNA-seq (*e.g.*, Smart-seq [22]). Previous studies have demonstrated that cells from different genetic backgrounds possess distinct mitochondrial DNA (mtDNA) haplotypes, which can be utilized for demultiplexing [20,23–25].

**Figure 1:**
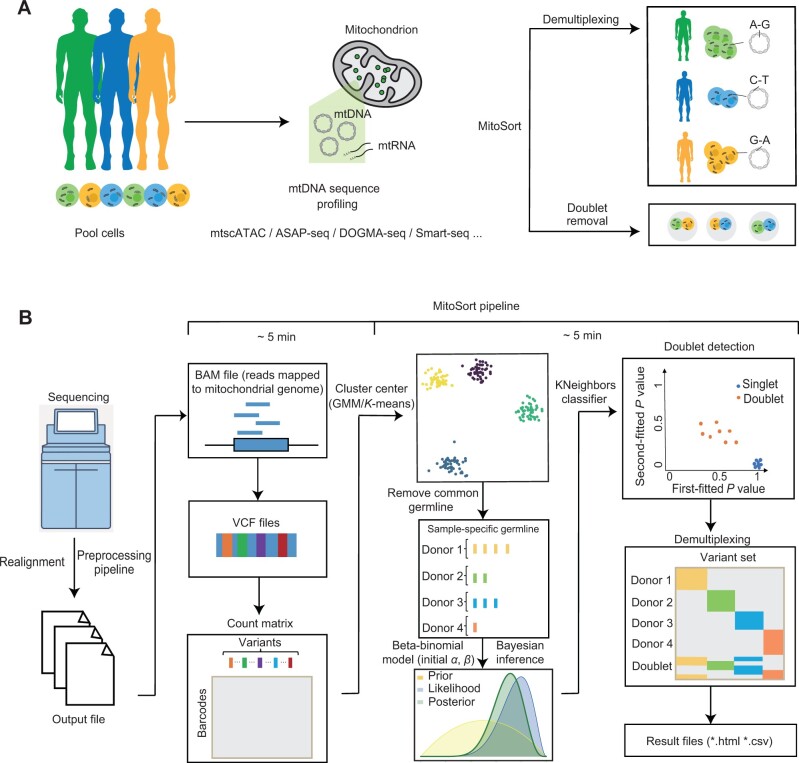
Workflow of MitoSort **A**. Schematic showing the purpose of MitoSort. **B**. Overview of MitoSort pipeline. Firstly, mitochondrial reads are realigned and putative SNPs are called. Subsequently, count matrices of reference and alternative alleles are generated for each cell, where each row represents a cell and each column represents a variant. Following that, sample-specific germline variants are identified for each individual. Cross-genotype doublets are then detected by a beta-binomial model. Lastly, singlets are demultiplexed for downstream analyses. SNP, single-nucleotide polymorphism; mtDNA, mitochondrial DNA; mtRNA, mitochondrial RNA; mtscATAC-seq, mitochondrial single-cell assay for transposase-accessible chromatin with sequencing; ASAP-seq, ATAC with select antigen profiling by sequencing; GMM, Gaussian Mixture Model.

Despite the benefits and feasibility of using mtDNA variants for demultiplexing samples and identifying doublets in single-cell sequencing data, especially in techniques that effectively profile mtDNA variants like mtscATAC-seq, few software tools are currently available for this purpose. To fill the gap, we developed MitoSort, a bioinformatic tool that exploits mtDNA germline variants exclusively to demultiplex samples from different individuals and detect cross-genotype doublets efficiently and accurately (**Figure 1**A). We evaluated the performance of MitoSort using *in silico* pooled and cell-hashed mtscATAC-seq data, demonstrating that MitoSort is a highly accurate and scalable method. MitoSort can also be applied to various single-cell sequencing approaches beyond mtscATAC-seq, provided that the mitochondrial sequence can be read out. Besides, the application of MitoSort to a case study further demonstrates that MitoSort can be used to accurately demultiplex samples of individuals under different pathologies, which facilitates downstream analyses without technical batch effects. Altogether, our results reveal the accuracy and efficiency of MitoSort, addressing the limitations that currently exist in computational techniques specifically tailored for scRNA-seq data. We believe that MitoSort will be a valuable tool, empowering efficient and reliable sample multiplexing in the realm of single-cell genomics applications for biomedical research.

## Method

### Overview of MitoSort

The MitoSort pipeline is mainly composed of four steps: mitochondrial read realignment, variant calling, doublet identification, and demultiplexing (Figure 1B). Among these, cross-genotype doublet identification and demultiplexing are the core objectives of MitoSort. Briefly, to reduce false-positive variants resulting from STAR [26] alignment artifacts, mitochondrial reads are realigned with GATK [27] to facilitate accurate variant calling. VarScan [28] is then used to identify putative single-nucleotide polymorphisms (SNPs) in mtDNA. An SNP-by-cell matrix is generated after filtering low-frequency variants. By the initial *K*-means clustering, sample-specific germline variants are identified, allowing for the precise clustering of cells based on unique mitochondrial haplotypes. To detect cross-genotype doublets, we model a cell’s allele counts as being drawn from a beta-binomial distribution, with parameters derived from one or two clusters. After the removal of cross-genotype doublets, singlet cells are assigned to their respective donor origin by assessing the probabilities associated with each cluster. Additionally, our model estimates the most likely number of pooled individuals, which is helpful when clinical information is lost.

### Detailed steps of MitoSort

#### Realignment for mitochondrial reads

To correct potential mapping errors around indels, the reads mapped to the mitochondrial genome were realigned locally using GATK v3.5 [27].

#### Variant calling and germline mutation selection

VarScan2 [28] was employed with the parameters “–min-var-freq 0.01, min-depth 8, and –min-reads2 2” to call mitochondrial variants using BAM files containing read pairs realigned to the mitochondrial genome. Since mitochondrial reads from the BAM file were contributed by multiple individuals, we retained variants with variant allele frequencies (VAFs) ranging from 1% to 99% as potential germline variants for each individual (1% < bulk VAF < 99%). To determine the number of reference alleles and alternative alleles in potential germline variants for each cell, we developed an in-house Python script (available at https://github.com/tangzhj/MitoSort) to generate two matrices, namely, Ref.matrix and Alt.matrix. For each potential germline variant per cell, the allele frequency was calculated as the ratio of alternative counts to all counts and stored in the Frequency.matrix. The Frequency.matrix contained the allele frequency of each potential germline variant, including sample-specific germline variants, common germline variants, and widely spread somatic variants.

To ensure that only germline variants were included in our analysis, a straightforward filtering criterion was employed. Specifically, we selected variants where the proportion of cells having a VAF above 99% among those with a non-zero VAF was higher than 50%. This criterion was chosen to ensure the selection of high-confidence germline variants with a high VAF in most cells, while simultaneously excluding low-coverage germline variants and somatic variants with low VAFs.

#### Identification of specific germline variants for each individual

To identify sample-specific germline variants while excluding common germline variants that could potentially interfere with subsequent doublet detection, we utilized the robust information on germline variants in each sample. First, we performed initial clustering for the Frequency.matrix using the *K*-means algorithm or Gaussian Mixture Model (GMM) from the sklearn library. The parameters used were “n_clusters = pooled_sample_number, max_iter = 1000, tol = 10^−5^, algorithm = ‘full’, n_init = 10”. Within each cell cluster, variants meeting the criterion where the ratio between cells with VAF > 99% and cells with VAF > 0 was higher than 0.5 were assigned as a variant set. Subsequently, we removed common variants that occurred in more than two sets to obtain the final set of specific germline variants for each individual. The approach allowed us to identify the cell clusters of each individual, as well as the sample-specific germline variants for the first time, providing an efficient and robust method for downstream analysis, such as doublet detection.

#### Cross-genotype doublet detection

After determining the initial clusters of cells or their corresponding cluster-specific germline variants, our next objective was to identify doublets between each cluster.

In our method, we define *K* as the number of clusters determined by the aforementioned steps. We also define *C* as the total number of cells, where the lower-case *c* is used for indexing and referring to specific cell barcodes. The number of sample-specific germline variant sets is denoted as *G*, with the lower-case *g* representing all the specific germline variants within a cluster, equivalent to *G_i_* (where *i* indicates cluster *i*). The notation *g,c* refers to the cluster-specific germline variants with observed data in cell *c*. Allele counts are represented by *A*. *A_g,c_* is a tuple of size 2, with the first number indicating the count of reference alleles and the second number indicating the count of alternative alleles observed in the cluster-specific germline variants in cell *c*.

For cluster-specific germline variants in each single cell, the observation of alternative allele counts follows a binomial distribution:

(1) PX=Ag,c,1=NAg,c,1pAg,c,11-pAg,c,0

where *p* is the probability of alternative allele frequency, *A_g,c,_*_1_ represents the alternative allele count, and *N* represents the total count for cluster-specific germline variants.

For a given cell, the probability of alternative alleles (*p*) or B-allele frequency (BAF) at sample-specific germline variants can be modeled by a Beta prior:

(2) fp=1Bα,βpα-11-pβ-1

The Beta prior model for *p* can be tuned to reflect the relative prior plausibility of each *p*.

Upon observing data *X* = *x* where *x* ∈ {0, 1, ···, *n*} and plugging *x* into the Binomial pmf Equation 1, the likelihood function of *p* is calculated as in Equation 3:

(3) LpX=Ag,c,1=NAg,c,1pAg,c,11-pAg,c,0

Applying Bayes’ Rule, the conjugate Beta prior combined with the Binomial data model produces a Beta posterior model for the posterior probability of cells belonging to each cluster (*p*). Putting Equation 2 and Equation 3 together, the posterior probability density function is described by Equation 4:

(4) fpX=Ag,c,1∝fpLpX=Ag,c,1∝pα+Ag,c,1-11-pβ+Ag,c,0-1

For each cluster, *α* and *β* can be estimated using the allele counts across all the sample-specific germline variants in one cluster. *α* = 1 + mean of alternative counts across variants in *G_i_* of *K_i_*, and *β* = 1.

For a cell *c* belongs to first-fitted cluster *i* (*K_i_*) and *g* = *G_i_*, *p_i_* is described by Equation 5:

(5) fpiX=Ag,c,1,Ag,c,1+Ag,c,0,c∈Ki,g=Gi∝Bα+Ag,c,1,β+Ag,c,0

When the cell *c* belongs to first-fitted cluster *i* (*K_i_*), but *g* = *G_j_*, *p*_*j*_ is described by Equation 6:

(6) fpjX=Ag,c,1,Ag,c,1+Ag,c,0,c∈Ki,g=Gj∝Bα+Ag,c,1,β+Ag,c,0

Then, *P_i_* is assigned as *p*_1_ for the first-fitted cluster, and the highest *P_j_* is selected as *p*_2_ for the second-fitted cluster. We estimate *p* as the mode of the posterior distribution. When the cell is a singlet, *p*_1_ will be dramatically higher than any *P_j_*.

To establish a reasonable threshold, we treat *p*_1_ and *p*_2_ as two-dimensional data. We categorize cells with *p*_1_ > 0.99 and *p*_2_ < 0.01 as “Singlet”, while cells with *p*_1_ < 0.8 or *p*_2_ > 0.2 as “Doublet” by default. This cutoff can be set by the user. To predict the class of unlabeled cells, we use the ‘KNeighborsClassifier’ method, which calculates the distance between the labeled cell point and all other unlabeled cell points in the dataset. The *k* = 5 nearest neighbors are identified based on the distance metric, and the majority class of the *k* = 5 nearest neighbors is used to predict the class of the unlabeled cells. It should be noted that if *p*_1_ is greater than 0.99, we recommend not considering *p*_2_, as the value of *p*_2_ may be caused by potential somatic mutations.

#### Demultiplexing pooled samples

After removing cross-genotype doublets identified in the previous step, individual cells are assigned to a cluster by the highest *p*. If cells are difficult to cluster initially under unsupervised clustering due to factors such as sequencing depth and highly imbalanced mixture proportions, we will cluster the germline variants and calculate probability values, refer to the MitoSort ‘--direct’ method. In cases where the exact number of pooled samples is unknown, we also provide the silhouette score method to estimate the optimal number of clusters. The silhouette score, which reaches its global maximum at the optimal number of clusters, enables accurate demultiplexing of pooled samples.

### Simulation of *in silico* data

In this study, we simulated mixtures of mtscATAC-seq data obtained from the 10X Genomics platform using custom Python scripts. Following the alignment of reads to the human reference genome hg38 using 10X Genomics Cell Ranger ATAC [29], mitochondrial reads were merged into a BAM file with cell barcodes prefixed to distinguish individuals. Next, reads were realigned using GATK [27]. In total, we obtained 52,283 cells across 8 samples. To systematically assess the performance of MitoSort, we generated synthetic mixtures into BAM files by random sampling using a range of parameter choices, including the number of reads mapped to mitochondrial genome (ranging from 250 to 2000), the number of pooled samples (ranging from 2 to 8), the number of cells for each sample (ranging from 100 to 600), and the percentage of doublets (1%, 5%, 10%, and 15%). Doublets were simulated by randomly choosing two cell barcodes and merging their read counts as a new cell. For each parameter set, simulations were repeated five times to account for variability.

### Benchmark against existing tools

We compared the performance of MitoSort against Souporcell [15], Vireo [14], and Freemuxlet (https://github.com/statgen/popscle) using simulated BAM files. Unless specified otherwise, default pipelines and recommended parameters were employed, and the number of threads was set to 8. For Souporcell, the *souporcell_pipeline.py* script was run within the singularity container with parameters “--no_umi True --min_alt 2 --min_ref 2”. For Vireo, cellsnp-lite was used to genotype a list of variants in each cell. Parameters used were “--minMAF 0.1 --minCOUNT 20 --UMItag None”. Subsequently, Vireo was utilized to demultiplex cells without any prior knowledge of genotypes. For Freemuxlet, the popscle dsc-pileup command was run within the singularity container provided by Demuxafy [30] to identify the number of reads from each allele at known variants with at least 1% minor allele frequency. Next, the popscle freemuxlet command was run to demultiplex cells. The effectiveness of each tool was assessed by comparing the classification outcomes with the true labels.

### Evaluation of MitoSort using cell hashing data

For the multiplexed ASAP-seq dataset comprising natural killer (NK) cells from four donors [31], we obtained a BAM file containing mitochondrial reads and hashtag oligonucleotide (HTO) count data for ASAP5 library downloaded from Gene Expression Omnibus (GEO: GSM6413442) upon request from the corresponding author of the published paper. HTO count data containing hashtag reads were used to assign each cell a hashtag ID by the HTODemux function within Seurat [32] package using default parameters. Subsequently, MitoSort was used to identify doublets and demultiplex samples based on the BAM file. Finally, we compared the donor assignment results obtained from MitoSort and those derived from the hashtag data.

For the multiplexed DOGMA-seq dataset comprising activated and stimulated T cells from two healthy donors [33], we first downloaded its HTO count data from the Gene Expression Omnibus (GEO: GSM6032896). Additionally, we obtained ATAC raw data from the NCBI Sequence Read Archive (SRA: SRX14779147). HTO count data containing hashtag reads were used to assign each cell a hashtag ID by the HTODemux function within Seurat [32] package. Raw reads in ATAC data were aligned to the human reference genome (hg38) using Cell Ranger ATAC [29]. Based on the generated BAM file, we ran MitoSort pipeline, including mitochondrial read realignment, variant calling, doublet identification, and demultiplexing. We compared cell barcodes classified as singlets by hashtag and MitoSort to evaluate the consistency of assignment results obtained from both approaches.

### Analysis of multiplexed Smart-seq3xpress data

We obtained raw data in the form of unmapped BAM files for two multiplexed Smart-seq3xpress datasets [34] from ArrayExpress (run accession Nos. ERR8607752 and ERR8607757). These two datasets contained cells from two and three donors, respectively. For each dataset, the unmapped BAM file was further processed using zUMIs (https://github.com/sdparekh/zUMIs). This involved mapping to the human reference genome (hg38) and quantification of gene expression. Cells that passed the quality control criteria, as stated in the original paper [34], were retained for downstream analysis. To expedite the demultiplexing process, 20% of reads in the BAM file were randomly sampled and used as input for MitoSort, Souporcell, and Vireo, respectively. The donor assignment results from each tool were compared against the cell identities derived from dual indexes, serving as the ground truth labels, to assess the performance of each tool. Besides, we generated simulated BAM files with varying numbers of reads per cell and compared the runtime of each tool.

### Isolation of PBMCs and B cells

To obtain peripheral blood mononuclear cells (PBMCs), the peripheral blood was diluted 1:1.5 in 0.9% physiological saline, and cells were isolated with a Lymphoprep density gradient (density 1.077 g/ml) according to the manufacturer’s instructions (Catalog No. 07851, STEMCELL, Vancouver, Canada). B cells were isolated using Miltenyi CD19 MicroBeads (Catalog No. 130-050-301, Miltenyi Biotec, Bergisch Gladbach, Germany) as described by the manufacturer. Cells were counted using Trypan Blue (Catalog No. 15250061, Thermo Fisher Scientific, Waltham, MA) exclusion under a hemocytometer.

### Single-cell multi-omic library construction and sequencing

Single-cell multi-omic libraries were generated using the 10X Chromium Controller and the Chromium Next GEM Single Cell Multiome ATAC + Gene Expression kit (Catalog No. PN-1000284, 10X Genomics, Pleasanton, CA) according to the manufacturer’s instructions. DNA LoBind tubes (Catalog No. 0030108051, Eppendorf, Hamburg, Germany) were used to wash cells in phosphate buffer saline (PBS) in downstream processing steps. To retain mtDNA, permeabilization was performed using low-loss lysis conditions, as described in mtscATAC-seq [20,21] with 10 mM Tris-HCl pH 7.4 (Catalog No. T2194, Sigma, Darmstadt, Germany), 10 mM NaCl (Catalog No. 59222C, Sigma), 3 mM MgCl_2_ (Catalog No. M1028, Sigma), 0.1% NP40 (Catalog No. 74385, Sigma), and 1% bovine serum albumin (BSA; Catalog No. 130-091-376, Miltenyi Biotec). After washing, cells were fixed with 1% formaldehyde (Catalog No. 28906, Thermo Fisher Scientific) in PBS for 10 min at room temperature, followed by quenching with glycine solution (Catalog No. G7126-500g, Sigma) to a final concentration of 0.125 M. After washing twice in PBS via centrifugation at 400 *g* for 5 min at 4°C, cells were treated with lysis buffer (10 mM Tris-HCl pH 7.4, 10 mM NaCl, 3 mM MgCl_2_, 0.1% NP40, 1% BSA) for 3 min. This was followed by adding 1 ml of chilled wash buffer (10 mM Tris-HCl pH 7.4, 10 mM NaCl, 3 mM MgCl_2_, 1% BSA) and inversion before centrifugation at 400 *g* for 5 min at 4°C. The supernatant was discarded, and cells were diluted in 1× Nuclei buffer (Catalog No. 2000207, 10X Genomics) before counting using Trypan Blue and a Countess II FL Automated Cell Counter. Briefly, after tagmentation, cells were loaded on a Chromium Controller Single-Cell instrument to generate single-cell Gel Bead-In-Emulsions (GEMs) followed by PCR as described in the 10X protocol. The final libraries were quantified using a Qubit dsDNA HS Assay kit (Catalog No. Q33231, Invitrogen, Carlsbad, CA) and a High Sensitivity DNA chip run on a Bioanalyzer 2100 system (Catalog No. 5067-4626, Agilent, Palo Alto, CA). Libraries were sequenced on Illumina NovaSeq 6000 at Berry Genomics.

### Analysis of multi-omic data of multiplexed B cells

Raw multi-omic data were preprocessed and aligned to the human reference genome (hg38) using the 10X Genomics Cell Ranger arc. Subsequently, the resulting BAM file from the ATAC data was subjected to the MitoSort pipeline for doublet identification and sample demultiplexing. Concurrently, the BAM file from the RNA data was processed using the Souporcell pipeline for doublet identification and sample demultiplexing. Furthermore, we utilized mitoSplitter [35] for demultiplexing, using the BAM file from the ATAC data as input. However, since mitoSplitter requires a mitochondrial variant allele frequency matrix file or bulk sequencing mapping BAM files for all samples as input, we partitioned the BAM file from the ATAC data into individual-specific BAM files. This partitioning was based on the donor assignment results obtained from Souporcell. These individual-specific BAM files were then used as input for mitoSplitter. Additionally, given that mitoSplitter is specifically designed for demultiplexing scRNA-seq data rather than scATAC-seq data, we modified its encapsulated code to skip the remapping of scRNA-seq data and instead directly provided the remapped BAM file.

The cell-by-gene and cell-by-peak count matrices were further processed using the R packages Seurat [32] and Signac [36]. We retained high-quality cells for downstream analysis based on the following criteria: fewer than 5000 RNA molecules, mitochondrial RNA content below 10%, 200–3500 expressed genes, 2000–50,000 ATAC fragments, transcription start site (TSS) enrichment score at least 5, nucleosome signal less than 1, and at least 50% of reads in peaks. Subsequently, weighted nearest neighbor (WNN) analysis was performed on both RNA and ATAC-seq modalities, resulting in 10 clusters. Cells were then classified into four distinct cell types using the following marker genes: B cells (*BANK1*, *MS4A1*), plasma cells (*MZB1*), T cells (*CD3D*, *CD3E*), and monocytes (*CD14*, *S100A9*). After removing cross-genotype doublets assigned by MitoSort, we obtained a total of 12,943 B cells. Next, WNN analysis was applied to the subclustering analysis of multi-omic data of B cells, which yielded five clusters. B cells were further divided into two subtypes using the following marker genes: naive B cells (*TCL1A*, *IL4R*) and memory B cells (*CD27*). Based on the donor origins of cells obtained from MitoSort, the percentage of each cluster in each sample was calculated.

## Results

### Evaluation of MitoSort using *in silico* data

To assess the performance of MitoSort, we first used *in silico* data with known cell origins to evaluate its accuracy and efficacy. We collected eight published mtscATAC-seq datasets from genetically distinct donors (detailed information is shown in Table S1). We sampled nuclear and mitochondrial reads per cell and created synthetic mixtures of cells from different donors, with 8% of the cells simulated as cross-genotype doublets (see Method).

**Figure 2:**
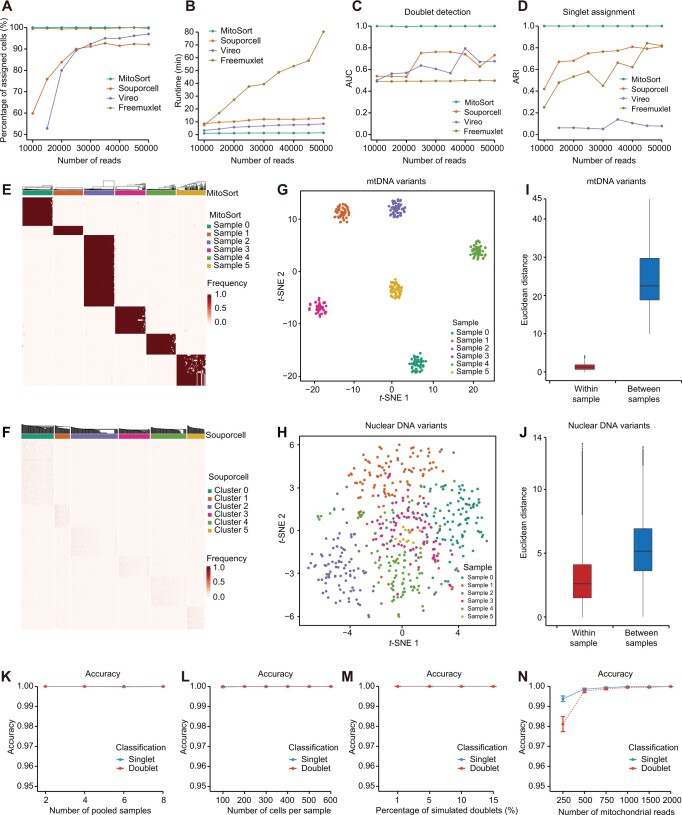
Evaluation of MitoSort using *in silico* data **A**.–**D**. Curves showing the percentage of assigned cells (A), runtime (B), AUC for doublet identification (C), and ARI between the true and inferred singlet assignment (D) for each tool on simulated data with 8 pooled samples (100 cells each) and varying reads per cell. **E**. and **F**. Heatmaps of sample-specific mitochondrial (E) and nuclear genome (F) germline variant frequencies across assigned singlets in simulated data (8 pooled samples, 100 cells per sample, 5000 reads per cell). The top legends show donor assignment by MitoSort (E) and Souporcell (F), respectively. **G**. and **H**. *t*-SNE plots of cell barcodes based on mitochondrial (G) and nuclear genome (H) germline variant profiles, colored by true sample origin. **I**. and **J**. Bar plots showing the distributions of Euclidean distances among cells within each sample and between samples, using sample-specific profiles of mitochondrial germline variants (I) and nuclear genome germline variant profiles (J). **K**.–**N**. Classification accuracy of MitoSort on simulated data with varying parameters: the number of pooled samples (K), the number of cells per sample (L), the percentage of simulated doublets (M), and the number of mitochondrial reads per cell (N). Five replicate runs were used to ensure the consistency of the results. AUC, area under the curve; ARI, adjusted Rand index; *t*-SNE, *t*-distributed stochastic neighbor embedding.

Current computational tools are mainly designed for demultiplexing scRNA-seq data using nuclear genome variants. Although some of them can be adapted to process mtscATAC-seq data, their performance in this context has not been systematically evaluated thus far. We applied MitoSort along with three existing computational tools, including Souporcell [15], Vireo [14], and Freemuxlet [12], to the synthetic datasets (8 pooled samples; 100 cells per sample with 8% of cells as doublets; and the number of mapped reads per cell ranged from 10,000 to 50,000) and subsequently compared their performance. We found that a significant proportion of cells could not be assigned to their respective donors when using Souporcell and Vireo (**Figure 2**A). Although there was an increase in the proportion of assigned cells with higher numbers of mapped reads per cell, their performance remained inferior to that achieved by MitoSort. Besides, despite consuming more than eight times the running time of MitoSort (Figure 2B), these tools failed to accurately identify cross-genotype doublets and assign singlets to their corresponding original donors (Figure 2C and D, Figure S1).

These results reveal the inadequate performance of computational tools relying on genome variants when applied to mtscATAC-seq data. We hypothesize that previous computational tools designed for scRNA-seq data require higher sequencing depth of nuclear genome to accurately assign cells. The sparsity of nuclear counts in mtscATAC-seq data makes it challenging to achieve this goal. To depict the genomic features of mtscATAC-seq data, we further quantified the characteristics of nuclear genome variants and mtDNA variants using the synthetic dataset with 50,000 mapped reads per cell. Firstly, the coverage of nuclear genome variants in mtscATAC-seq data was significantly lower than that of mtDNA variants (Figure S2A–C). Secondly, the frequency of sample-specific nuclear genome variants among cells from the same samples exhibited lower consistency and robustness when compared to mtDNA variants (Figure 2E and F). Consequently, the discriminatory power of nuclear genome variants in distinguishing cells from different samples is inferior to that of mtDNA variants (Figure 2G–J). In light of these observations, it becomes evident that mtDNA variants serve as superior endogenous genetic barcodes for mtscATAC-seq data. MitoSort, which takes advantage of the characteristics of mtDNA, can address the shortcomings of previous computational tools and offer improved functionality in demultiplexing mtscATAC-seq data.

We further assessed the impact of factors affecting the classification performance of MitoSort. Among these factors, the number of pooled samples, the number of cells per sample, and the percentage of simulated doublets had little effect on the classification performance of MitoSort (Figure 2K–M, Figure S3A–F). We consistently obtained high accuracy in donor assignment and doublet identification for 2 to 8 pooled samples (500 cells per sample, 2000 mtDNA reads per cell, and 8% of cells as doublets) (Figure 2K). Besides, when we synthetically mixed 8 samples (2000 mtDNA reads per cell and 8% of cells as doublets) and varied the number of cells from 100 to 600 per sample, we found that the true-positive rates (TPRs) of both donor assignment and doublet identification were consistently achieved at a rate of 1 (100%), even when the number of cells was as low as 100 (Figure S3C). Only a small number of singlets was erroneously classified as doublets, constituting less than 0.3% of all cases (Figure S3D). In addition, MitoSort achieved perfect performance regardless of the percentage of simulated doublets (Figure 2M, Figure S3E and F). We found that the performance of MitoSort was only sensitive to the sequencing depth of mitochondrial genome (Figure 2N). However, even with as few as 250 mapped reads of the mitochondrial genome, MitoSort achieved an accuracy exceeding 97%. Specifically, an increase in the number of mapped reads (ranging from 250 to 2000) resulted in a higher TPR and a lower false discovery rate (FDR), leading to improved accuracy (Figure 2N, Figure S3G and H). It is worth noting that when the number of reads mapped to the mitochondrial genome reached approximately 1000 (representing roughly 4×), around 85% of sample-specific mtDNA variants can be well captured (Figure S4), and MitoSort was able to achieve perfect cell assignment (100% accuracy) to their respective donors and accurately detect cross-genotype doublets (Figure 2N). Collectively, these data show that MitoSort is a highly accurate and robust method for demultiplexing mtscATAC-seq data derived from distinct individuals and identifying cross-genotype doublets using mtDNA germline variants.

### Evaluation of MitoSort using pooled datasets with cell hashtags

**Figure 3:**
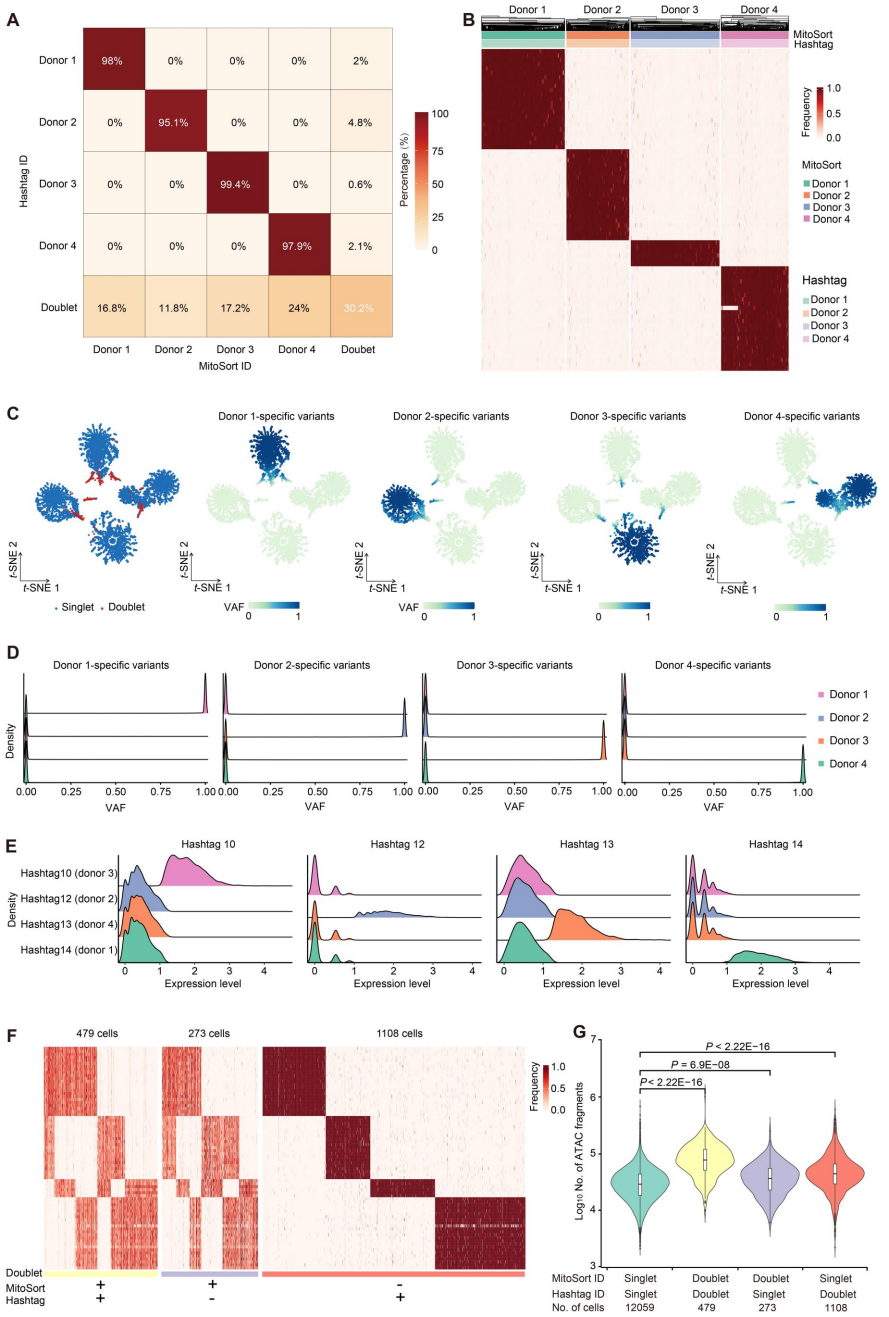
Evaluation of MitoSort using cell hashing data **A**. Percentage of barcodes shared between hashtag-based (rows) and MitoSort-based (columns) assignments in multiplexed ASAP-seq data consisting of four donors. **B**. Heatmap showing the allele frequency of sample-specific mitochondrial germline variants (rows) across singlets (columns) in multiplexed ASAP-seq data. The top legend shows the donor assignments of MitoSort and the hashtag-based approach. **C**. *t*-SNE plots of cell barcodes by the frequency of sample-specific mitochondrial germline variants, colored by singlet/doublet classification (left) or by average frequency for the donor-specific mitochondrial germline variants (four right panels). **D**. Distributions of sample-specific germline variant frequency for each donor across four singlet groups assigned by MitoSort. **E**. Distributions of counts for each hashtag across the four hashtag-assigned groups. **F**. Heatmap showing the allele frequency of sample-specific mitochondrial germline variants (rows) across cells (columns) for concordant and discordant doublets. **G**. Distribution of the number of ATAC fragments per cell in four groups with concordant or discordant assignments. *P* values were calculated using the Wilcoxon rank-sum test. VAF, variant allele frequency.

Cell hashing with oligo-tagged antibodies allows assigning sample origins to cells in mtscATAC-seq datasets based on the counts of HTOs [11]. Using the HTO-based assignment as ground truth, we next evaluated our method with two published experimentally multiplexed datasets using the cell-hashing method. For the ASAP-seq dataset comprising NK cells from four donors [31], MitoSort demonstrated high consistency (∼ 98%) in donor identity assignment (**Figure 3**A, Figure S5A). Singlets originating from the same donor exhibited identical mitochondrial genotypes (Figure 3B and C, Figure S5B). Compared to hashtag counts, the bimodal distributions of allele frequency for donor-specific mitochondrial germline variants exhibited more pronounced disparities among different singlet groups (Figure 3D and E). It reveals that mtDNA variants can serve as a more computationally efficient and robust feature for donor assignment. For doublet identification, we observed that the hashtag-based approach identified more doublets than MitoSort, resulting in a notable proportion of cells with inconsistent singlet/doublet classifications between the two strategies (Figure 3A). Upon examining the mitochondrial haplotypes of these discordant cells, we observed that cells that were classified as doublets solely by MitoSort indeed contained multiple mitochondrial genotypes, whereas cells that were classified as doublets solely by hashtag-based approach contained only one mitochondrial genotype (Figure 3F). We next compared the numbers of chromatin fragments in the three doublet groups with that of the singlet group. We found that the doublets defined by the two methods exhibited a significantly higher sequencing depth compared to the singlet group, while the doublets defined only by MitoSort (the Doublet-Singlet group) showed a slightly higher sequencing depth (Figure 3G). Encouraged by the clarity of mitochondrial genotype (Figure 3F), we further examined the original counts of hashtags for the cells in the Doublet-Singlet group. We found that the HTO demultiplexing tool tended to assign cells with higher HTO counts as doublets (Figure S6A and B). The cells in the Doublet-Singlet group exhibited dual HTO tags; however, their counts were comparatively lower, resulting in the false-negative identification of doublets by the hashtag-based method (Figures S6C–E and S7A–C). Interestingly, the number of chromatin fragments in doublets called only by hashtag-based approach (the Singlet-Doublet group) was also higher than that of the singlet group (Figure 3G). It suggests that these cells may be doublets from the same genotype, which cannot be distinguished by MitoSort. Given that this dataset comprises four individuals (*N* = 4) and assumes an equal mixing of cells from different individuals, the probability of doublets contributed by the same genotypes is 1/4, while the probability of doublets containing cells from different individuals is 3/4. With the detection of 479 Doublet-Doublets and 273 Doublet-Singlets (Figure 3F and G), we determined that there were 752 doublets originating from different genotypes. Since these cross-genotype doublets accounted for 3/4 of the total doublets, we deduced that the number of doublets contributed by the same genotypes was 250 [calculated as 752 divided by (3/4) times (1/4)]. This implies that the proportion of doublets contributed by the same genotypes is estimated to be 1.8% of total cells (250 cells out of a total of 13,919 cells). However, the proportion of observed cells in the Singlet-Doublet group was about 8% (1108 cells), which was much higher than the expected number. It indicates that there is false-positive calling by hashtag-based approach (Figures S6C–E and S7A–C). In addition to its accuracy, MitoSort also showed higher sensitivity compared to the hashtag-based approach. MitoSort was able to assign donor and singlet/doublet status for 99.1% of the cells, compared to only 62.4% achieved by the hashtag-based approach (Figure S7D and E). These findings indicate that the performance of demultiplexing by exogenous hashtags is heavily dependent on the outcome of hash-tagging experiment, while endogenous germline mitochondrial variants are more stable and could be used for experimental efficiency evaluation and quality control measures.

MitoSort also achieved great performance on a DOGMA-seq dataset which consisted of activated and stimulated T cells from two healthy donors [33]. Notably, when the sequencing depth of mitochondrial genome was not less than 4×, MitoSort consistently achieved a high level of accuracy in assigning cellular origins, with an accuracy rate of no less than 99% (Figure S8A and B). Collectively, these results further suggest the robustness of MitoSort in assigning cells from different donors and identifying cross-genotype doublets using mtDNA germline variants.

### Application of MitoSort to pooled full-length scRNA-seq datasets

**Figure 4:**
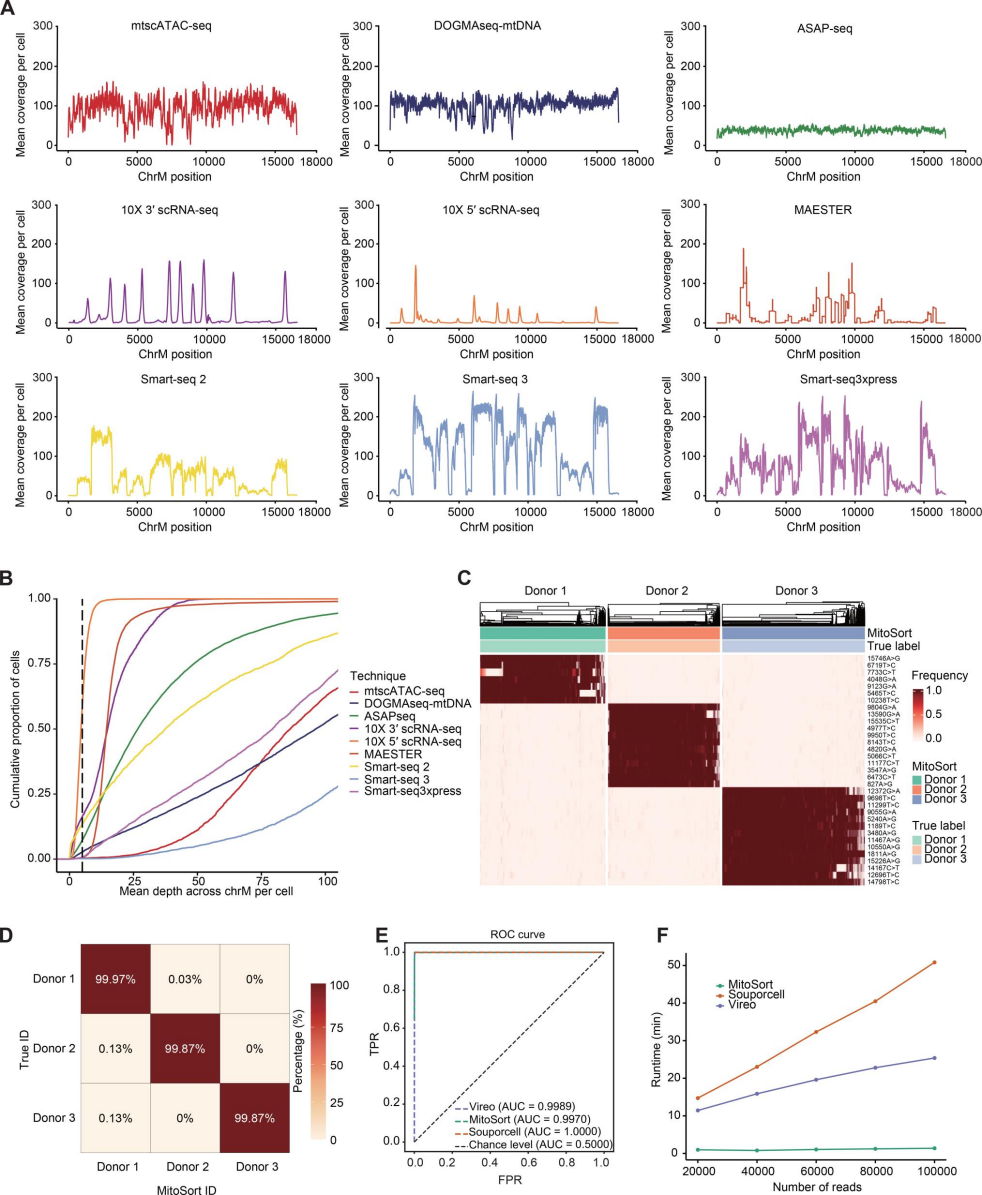
Extension of MitoSort to full-length scRNA-seq data **A**. Coverage comparison across three scATAC-seq datasets and six scRNA-seq datasets. Droplet-based scRNA-seq data show an uneven distribution of reads across the mitochondrial genome while others are generally more well-covered. **B**. An ECDF plot showing average sequencing depth across the mitochondrial genome per cell for nine datasets using different sequencing techniques. **C**. Heatmap showing the allele frequency of sample-specific mitochondrial germline variants (rows) across MitsoSort-assigned singlets (columns) in the multiplexed Smart-seq3xpress dataset. The top legend shows the donor assignments obtained from MitoSort and dual indexes. **D**. Percentage of barcodes shared between dual index-based (rows) and MitoSort-based (columns) assignments in the multiplexed Smart-seq3xpress data consisting of three donors. **E**. ROC curves for singlet assignment of each tool in the multiplexed Smart-seq3xpress data consisting of three donors. **F**. Runtime of each tool in the simulated Smart-seq3xpress data containing three donors, each with 200 cells and varying numbers of reads per cell. ECDF, Empirical Cumulative Distribution Function; ROC, receiver operating characteristic; TPR, true-positive rate; FPR, false-positive rate.

We have demonstrated that MitoSort is a highly accurate and scalable method for single-cell sequencing protocols in which mitochondrial genome could be efficiently captured. Previous studies have demonstrated that mitochondrial variants can also be captured in scRNA-seq protocols with high coverage [23,37]. We next evaluated the coverage and capture rate of mitochondrial genome in nine single-cell sequencing techniques (**Figure 4**A and B). Compared to droplet-based scRNA-seq techniques that typically sequence the 3′ or 5′ ends of RNA molecules, full-length scRNA-seq protocols offer improved coverage of mitochondrial transcripts and facilitate variant calling for larger parts of the mitochondrial genome (Figure 4A). In each full-length scRNA-seq dataset, approximately over 90% of cells achieved an average sequencing depth of more than 5× across the entire mitochondrial genome (Figure 4B, Figure S9A). This demonstrates the feasibility of utilizing MitoSort to demultiplex full-length scRNA-seq data as well.

We next evaluated the performance of MitoSort using the Smart-seq3xpress dataset, which consisted of cells from three donors [34]. The classification results obtained by MitoSort were highly consistent with true donor identities derived from dual indexes (Figure 4D). Cells assigned to the same donors shared identical mitochondrial haplotypes (Figure 4C). We also benchmarked our method against Souporcell and Vireo, two of the best computational tools for demultiplexing pooled scRNA-seq data using nuclear genome variants. MitoSort demonstrated comparable accuracy in singlet assignment to the tools specifically developed for scRNA-seq data (Figure 4E) and showed higher accuracy in doublet identification (Figure S9B). Furthermore, MitoSort exhibited better computational efficiency compared to other tools, with superior processing speed when applied to the same dataset (Figure 4F). We obtained similar results when applying MitoSort to another Smart-seq3xpress dataset comprising cells from two donors (Figure S9C and D). Collectively, these results demonstrate the versatility of MitoSort. It can be employed for accurately demultiplexing pooled datasets derived from various single-cell genomic libraries, provided that the mitochondrial sequence can be appropriately read out.

### Application of MitoSort to a single-cell multi-omic dataset pooled from eight donors

Due to the high cost of single-cell genomics approaches, conducting technical replicates is often unfeasible for most of the current studies. However, the presence of batch effects can diminish the sensitivity of identifying true biological signals and may introduce artifacts. Demultiplexing samples within the same experimental run can effectively reduce artifacts arising from sample preparation and library construction, enhancing the accuracy and reliability of downstream analyses.

**Figure 5:**
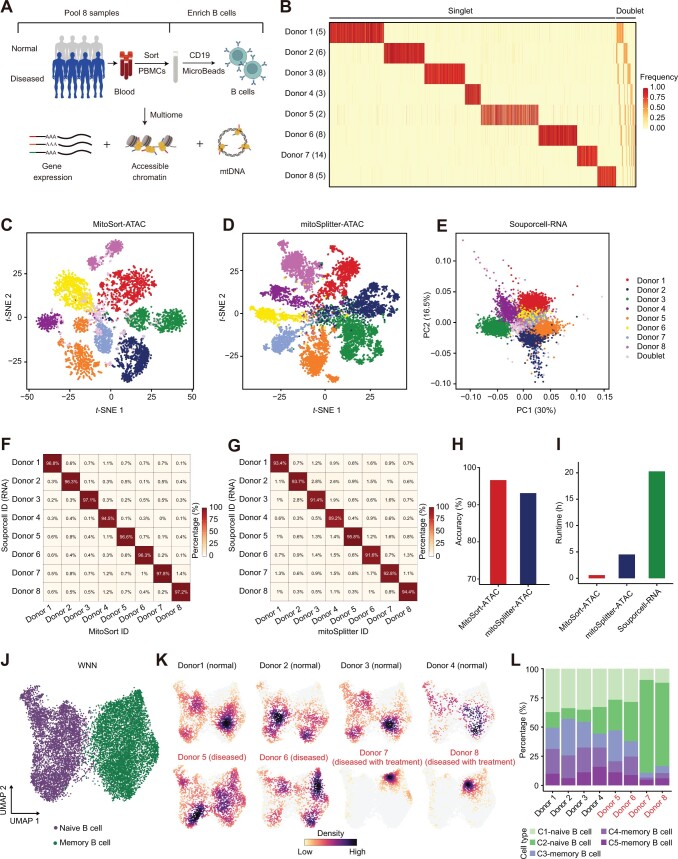
Multi-omic analysis based on MitoSort-identified depletion of B cells in RTX-treated patients **A**. Schematic of experimental design. PBMCs from four normal individuals and four patients with autoimmune diseases were collected and pooled. Next, B cells were enriched to profile gene expression, chromatin accessibility, and mtDNA. **B**. Heatmap showing the allele frequency of aggregated sample-specific mitochondrial germline mutations (rows) across cells (columns) sorted by MitoSort assignment. The number in the parenthesis of each row name represents the number of sample-specific mitochondrial germline mutations for this sample. **C**. and **D**. *t*-SNE plots of cell barcodes based on sample-specific mitochondrial germline variant profiles obtained from MitoSort (C) and mitoSplitter (D). Each dot represents a cell barcode and is colored by the cell classification of MitoSort (C) or mitoSplitter (D). **E**. PCA plot of the normalized cell-by-cluster log-likelihood matrix from Souporcell. Each dot represents a cell barcode and is colored by the cell classification of Souporcell. **F**. and **G**. Percentage of barcodes shared between Souporcell-based and MitoSort-based (F) or mitoSplitter-based (G) assignments in multiplexed multi-omic data consisting of eight donors. **H**. Bar plot showing the accuracy of cell donor assignment using different methods. Accuracy for each method is computed by considering Souporcell-based donor assignment as true labels. **I**. Runtime of different demultiplexing methods. **J**. UMAP of WNN graph for RNA and ATAC-seq modalities showing cell type annotation for each cell. **K**. UMAP of WNN graph for RNA and ATAC-seq modalities showing cells per individual colored by cell number densities. Disease status and clinical information for each individual are shown in subtitles. **L**. Bar plot showing the percentage of each cell cluster in each individual. RTX, rituximab; PBMC, peripheral blood mononuclear cell; PCA, principal component analysis; PC, principal component; UMAP, Uniform Manifold Approximation and Projection; WNN, weighted-nearest neighbor.

We conducted a case study in which we multiplexed B cells from eight individuals to profile gene expression, chromatin accessibility, and mtDNA (**Figure 5**A, Figure S10A–C) in one library. The ATAC assay contained an average of 25% mtDNA reads and 19× mtDNA coverage per cell, which allowed for accurate mitochondrial variant calling and demultiplexing (Figure S10D and E). Using MitoSort, we could clearly classify a total of 16,472 cells into 8 singlet populations, as well as doublet groups based on the allele frequency of individual-specific mitochondrial germline variants (Figure 5B). In addition, 6.4% of cell barcodes were identified as cross-genotype doublets involving more than one mitochondrial genotype (Figure 5B and C). Furthermore, we employed mitoSplitter [35] for demultiplexing based on the ATAC assay in multi-omic data (Figure 5D). mitoSplitter is specifically designed for demultiplexing scRNA-seq datasets using mtDNA variants. However, unlike MitoSort, it requires reference SNP information obtained from bulk sequencing for each sample as input, which is often unavailable. Given the absence of true labels in this real pooled dataset, we also used Souporcell for demultiplexing based on the RNA assay in multi-omic data (Figure 5E) and compared its outcomes with those of MitoSort and mitoSplitter (Figure 5F and G). Our analysis revealed that even when mitoSplitter was provided with sample-specific mitochondrial variants as input, MitoSort exhibited higher consistency (96.6%) with Souporcell in donor identity assignment compared to mitoSplitter (93.1%) (Figure 5H). This underscores MitoSort’s superior performance in achieving high accuracy without requiring reference SNP information. Moreover, MitoSort exhibited remarkable computational efficiency, completing the demultiplexing process approximately 1/7th and 1/20th of the time taken by mitoSplitter and Souporcell, respectively (Figure 5I).

We further performed high-dimensional clustering of the multi-omic profiles from 12,943 high-quality B cells, identifying 5 clusters. These clusters were annotated as naive B cells and memory B cells based on known markers (Figure 5J, Figure S10F and G). Among the 8 individuals, we found that the proportions of each B cell subtype were significantly different (Figure 5K and L). By utilizing mtDNA SNP information, we are able to match donors with MitoSort-assigned labels. According to the clinical records, the individuals with lower proportions of memory B cells had received rituximab (RTX) treatments, which was consistent with the function of RTX to deplete memory B cells (Figure 5K and L) [38]. It suggests that the observed differences in cell composition reflect true biological differences.

Collectively, these findings demonstrate that MitoSort’s donor assignment accuracy is superior to another mtDNA-based demultiplexing tool and is comparable to that of the state-of-the-art tool Souporcell in the same real pooled dataset. Moreover, MitoSort exhibits higher efficiency in demultiplexing time, which is crucial when applied to large-scale clinical studies [5]. MitoSort facilitates accurate donor demultiplexing and efficiently identifies their different pathologies at both a low experimental and computational costs.

## Discussion

In conclusion, the unique properties of mtDNA make it a more efficient and robust choice for endogenous genetic barcodes in sample demultiplexing and the detection of cross-genotype doublets. However, there are currently few software options available for this purpose. MitoSort fills this gap by providing an accurate and scalable method for demultiplexing pooled single-cell genomic data across donors and identifying cross-genotype doublets in various sequencing techniques, including mtscATAC-seq, ASAP-seq, DOGMA-seq, and Smart-seq. In our case study, where cells from eight donors were pooled, we demonstrate that only two sample-specific mitochondrial variants (donor 5) are sufficient to achieve an accuracy of 96.6%. Although the number of mitochondrial variants is limited compared to the nuclear genome, their coverage and allele frequency are more distinguishable. Nonetheless, the matrilineal inheritance of mitochondria poses a limitation to this approach, as it is challenging to distinguish individuals with haploidentical mtDNA, such as siblings of the same mother and mother-child pairs. Future tools may leverage both mitochondrial and nuclear variants to capitalize on their combined strengths and potentially achieve wider applicability in this field.

In this study, we systematically compared the demultiplexing performance of tools that utilize endogenous genetic variations to distinguish cells from different donors. These tools can be categorized based on two main aspects: (1) the type of sample markers used (nuclear or mitochondrial variants) and (2) whether they require a genotype reference obtained via whole-genome/exome sequencing. In many scenarios, a genotype reference or sample-specific mitochondrial variants are unavailable. Therefore, tools that do not require a genotype reference, such as Souporcell, Vireo, Freemuxlet, and MitoSort, may be prioritized. Additionally, the choice of demultiplexing method heavily depends on the specific data type. For droplet-based 3′ or 5′ scRNA-seq data, Souporcell is currently considered the state-of-the-art demultiplexing method. However, in the case of mtscATAC-seq data, MitoSort demonstrates superior accuracy and efficacy. Moreover, for full-length scRNA-seq data or single-cell multi-omic data containing both RNA and ATAC information, users can benefit from leveraging different tools and integrating diverse layers of information for demultiplexing. For instance, in our analysis of a single-cell multi-omic dataset where cells from eight donors were pooled, we utilized Souporcell for demultiplexing based on the RNA assay and MitoSort for demultiplexing based on the ATAC assay in multi-omic data. This integrated approach has the potential to enhance demultiplexing performance by combining results from both assays. It is also notable that MitoSort exhibits remarkable computational efficiency compared to other demultiplexing tools. In cases of large-scale research projects, the efficiency of MitoSort can provide a substantial advantage and help accelerate the research process. Furthermore, MitoSort’s shorter runtime may facilitate faster iterations and optimization, contributing to enhanced performance.

Our systematic comparison reveals that all the currently available methods for doublet detection have their own limitations and biases. Profiling-based methods, which rely on the transcriptional profiles for doublet detection [39], may not be effective in cases where the pooled cells lack cell type diversity. Genotyping-based methods, including MitoSort, fail to detect doublets originating from the same individual. Experimental-based methods for doublet detection rely on labeling efficiency. However, the advantage of genotyping-based methods is that as the number of pooled samples increases, the probability of doublet from the same individual decreases. We anticipate that genotyping-defined doublets could serve as valuable positive controls to guide the refinement of doublet detection through profiling-based methods, ultimately enhancing the accuracy of doublet identification in future studies.

## Ethical statement

The study protocol was approved by the local ethics committee of Sun Yat-sen University (Approve No. 2015/521), and written informed consent was obtained from all donors. All experiments and protocols involving human samples were conducted in accordance with relevant guidelines and regulations.

## Code availability

MitoSort pipeline and scripts for reproducing the study are available on GitHub (https://github.com/tangzhj/MitoSort) and BioCode (https://ngdc.cncb.ac.cn/biocode/tools/BT007502).

## Supplementary Material

qzae073_Supplementary_Data

## Data Availability

Single-cell multi-omic data generated in this study have been deposited in the Genome Sequence Archive for Human [40] at the National Genomics Data Center, Beijing Institute of Genomics, Chinese Academy of Sciences / China National Center for Bioinformation (GSA-Human: HRA004522), and are publicly accessible at https://ngdc.cncb.ac.cn/gsa-human.

## CRediT author statement

**Zhongjie Tang:** Data curation, Methodology, Software, Formal analysis, Visualization, Writing – original draft, Writing – review & editing. **Weixing Zhang:** Investigation, Resources. **Peiyu Shi:** Data curation, Software, Formal analysis, Visualization, Writing – original draft, Writing – review & editing. **Sijun Li:** Validation, Formal analysis, Visualization. **Xinhui Li:** Validation. **Yueming Li:** Validation. **Yicong Xu:** Validation. **Yaqing Shu:** Resources. **Zheng Hu:** Validation. **Jin Xu:** Conceptualization, Supervision, Funding acquisition, Writing – review & editing. All authors have read and approved the final manuscript.
