# Supplementary material for: MitoSort: Robust Demultiplexing of Pooled Single-cell Genomic Data Using Endogenous Mitochondrial Variants
Source: Genomics Proteomics Bioinformatics. 2024 Oct 15;22(5):qzae073. doi: 10.1093/gpbjnl/qzae073 (PMC11671100; doi:10.1093/gpbjnl/qzae073)
Supplement: qzae073_Supplementary_Data [file qzae073_supplementary_data.zip › supplementary material captions.docx]

**Supplementary material**

**Figure S1 ROC curves for doublet identification of each tool on simulated data**

The simulated data contained eight pooled samples, each with 100 cells and varying numbers of reads per cell.

**Figure S2 Sequencing depth of nuclear and mitochondrial germline variants in simulated data**

**A.** and **B.** Histograms showing the distributions of sequencing depth for sample-specific nuclear genome germline variants (A) and mitochondrial germline variants (B) per cell in simulated data containing eight pooled samples, each with 100 cells and 5000 reads per cell. **C.** An ECDF plot showing sequencing depth of sample-specific nuclear genome and mitochondrial germline variants in the same simulated data.

**Figure S3 Systematic assessment of MitoSort performance on simulated data using a range of parameter choices**

The TPR and FDR of donor assignment and doublet identification by MitoSort on simulated datasets with varying parameters: the number of mixed samples (**A**–**B**), the number of cells per sample (**C**–**D**), doublet rates (**E**–**F**), and the number of mitochondrial reads per cell (**G**–**H**). Unless specified, the defaults are 8 mixed samples, 500 cells per sample, an 8% doublet rate, and 2000 mitochondrial reads per cell. TPR, true positive rate; FDR, false discovery rate.

**Figure S4 Capture rates of sample-specific mitochondrial germline variants across varying mitochondrial reads**

**Figure S5 Performance of MitoSort in multiplexed ASAP-seq data across varying sequencing depths**

**A.** Percentage of barcodes with different sequencing depths of mitochondrial genome (5–10×, 10–20×, and > 20×) shared between hashtag-based (rows) and MitoSort-based (columns) assignments. **B.** Heatmaps showing the allele frequency of sample-specific mitochondrial germline mutations (columns) across cells (rows) with different sequencing depths of mitochondrial genome (5–10×, 10–20×, and > 20×).

**Figure S6 Comparison of MitoSort-based and hashtag-based results in cell hashing data**

**A.** Distribution of hashtag counts in four groups with concordant or discordant assignments. *P* values were calculated using the Wilcoxon rank-sum test. **B.** Correlation of the number of ATAC fragments and hashtag counts. **C.**–**E.** Scatter plots showing raw counts for pairs of hashtags, colored by hashtag-based assignment (left), or by combined assignment from MitoSort and hashtag-based approach (right).

**Figure S7 Further comparison of MitoSort-based and hashtag-based results in cell hashing data**

**A.**–**C.** Scatter plots showing raw counts for pairs of hashtags, colored by hashtag-based assignment (left), or by combined assignment from MitoSort and hashtag-based approach (right). **D.** Percentage of cell assignment of MitoSort and hashtag-based approach on multiplexed ASAP-seq data consisting of four donors. **E.** Read counts for each hashtag (rows) across cells (columns) sorted by their HTODemux classification.

**Figure S8 Performance of MitoSort in multiplexed DOGMA-seq data across varying sequencing depths**

**A.** Percentage of barcodes with different sequencing depth of mitochondrial genome (2-4×, 4-8×, and > 8×) shared between hashtag-based (rows) and MitoSort-based (columns) assignments. **B.** Heatmap showing the allele frequency of sample-specific mitochondrial germline variants (columns) across cells (rows) with different sequencing depth of mitochondrial genome (2-4×, 4-8×, and > 8×).

**Figure S9 Performance of MitoSort in multiplexed full-length scRNA-seq data**

**A.** An ECDF plot showing average sequencing depth across mitochondrial genome per cell for nine datasets using different sequencing techniques. **B.** Percentage of cell assignment of each tool in multiplexed Smart-seq3xpress dataset consisting of three donors. Both Souporcell and Vireo produce false positive results in doublet identification. **C.** Percentage of barcodes shared between dual indexes-based (rows) and MitoSort-based (columns) assignments in multiplexed Smart-seq3xpress data consisting of two donors. **D.** Heatmap showing the allele frequency of sample-specific mitochondrial germline variants (rows) across MitoSort-assigned singlets (columns) in multiplexed Smart-seq3xpress dataset consisting of two donors. The top legend shows the donor assignments obtained from MitoSort and dual indexes.

**Figure S10 Multi-omics data analysis of multiplexed B cells**

**A.** Violin plot showing the distribution for the number of RNA molecules per cell in the RNA assay of multi-omics data. **B.** Aggregated normalized accessibility around TSSs for the ATAC assay of multi-omics data. **C.** The TSS enrichment scores and fragment counts for each B cell. The dot color represents the density in arbitrary units of points in the plot. **D.** Violin plot showing the distribution for the percentage of mitochondrial fragments per cell in the ATAC assay of multi-omics data. **E.** Average sequencing coverage across the mitochondrial genome per cell in the ATAC assay of multi-omics data. **F.** UMAP of WNN graph for RNA and ATAC-seq modalities. Each dot represents a cell colored by its cluster. **G.** Dot plot showing the expression of marker genes in each cluster. Dot size indicates the proportion of cells expressing the gene, and dot color represents the scaled average expression level. TSS, transcription start site.

**Table S1 Sources of datasets used in the study**
