# Supplementary material for: MitoSort: Robust Demultiplexing of Pooled Single-cell Genomic Data Using Endogenous Mitochondrial Variants
Source: Genomics Proteomics Bioinformatics. 2024 Oct 15;22(5):qzae073. doi: 10.1093/gpbjnl/qzae073 (PMC11671100; doi:10.1093/gpbjnl/qzae073)
Supplement: qzae073_Supplementary_Data [file qzae073_supplementary_data.zip › Table S1.docx]

**Table S1 Sources of datasets used in the study**

| **Dataset** | **Library** | **No. of individuals** | **Usage** | **Ref.** |
| --- | --- | --- | --- | --- |
| GSE142745 | mtscATAC-seq | 5 | Simulation | [20] |
| GSE156477 | mtscATAC-seq | 1 | Simulation | [21] |
| GSE156474 | mtscATAC-seq | 1 | Simulation | [21] |
| GSE156478 | mtscATAC-seq | 1 | Simulation | [21] |
| GSM6413442 | ASAP-seq | 4 | Evaluation | [31] |
| GSM6032896 SRX14779147 | DOGMA-seq | 2 | Evaluation | [33] |
| ERR8607752 | Smart-seq3 | 1 | Evaluation | [34] |
| ERR8607757 | Smart-seq3 | 1 | Evaluation | [34] |
| PRJCA016782 | Single cell Multiome | 8 | Evaluation | This study |
