# Supplementary figures and images for: MitoSort: Robust Demultiplexing of Pooled Single-cell Genomic Data Using Endogenous Mitochondrial Variants
Source: Genomics Proteomics Bioinformatics. 2024 Oct 15;22(5):qzae073. doi: 10.1093/gpbjnl/qzae073 (PMC11671100; doi:10.1093/gpbjnl/qzae073)

**A**

Xu et. al DOGMA-seq (2-4×)

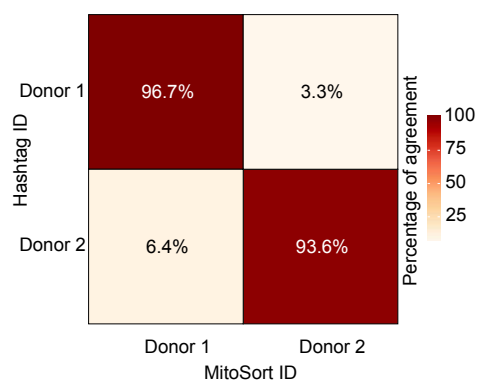

Xu et. al DOGMA-seq (4-8×)

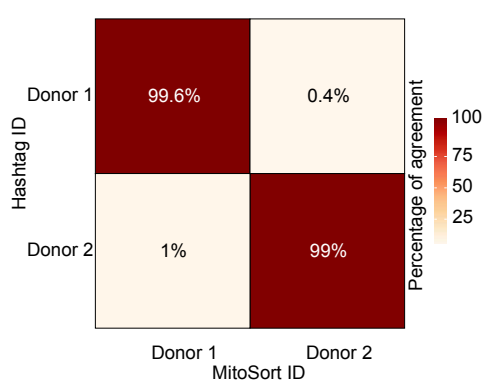

Xu et. al DOGMA-seq (&gt; 8×)

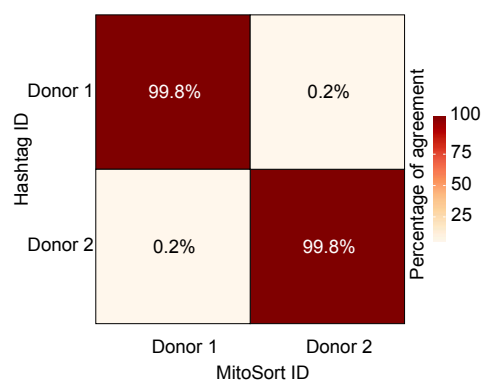**B**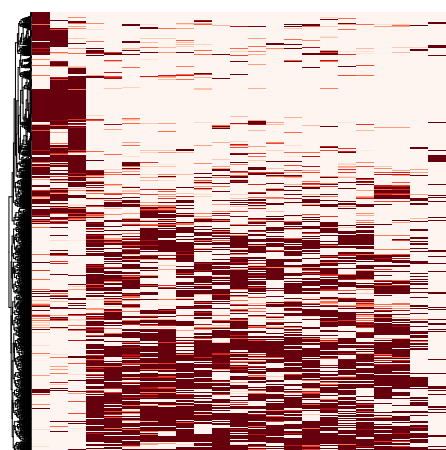

Frequency  
0 0.5 1

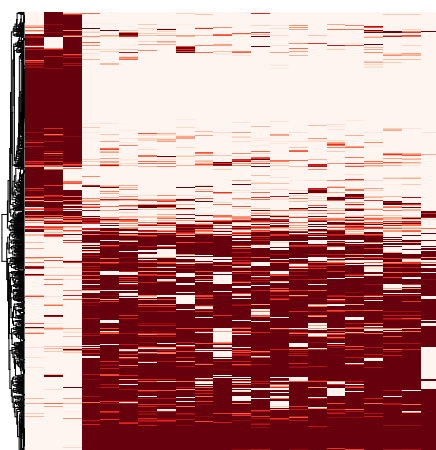

Frequency  
0 0.5 1

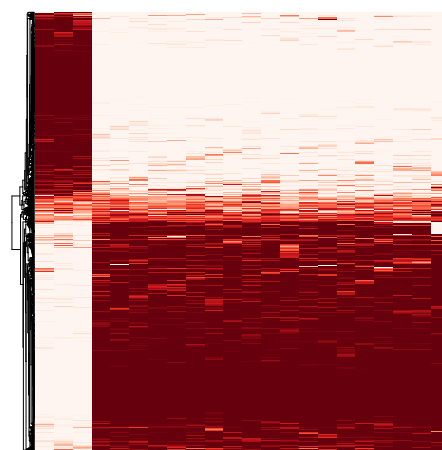

Frequency  
0 0.5 1

Supplement: qzae073_Supplementary_Data [file qzae073_supplementary_data.zip › Figure S8.pdf]

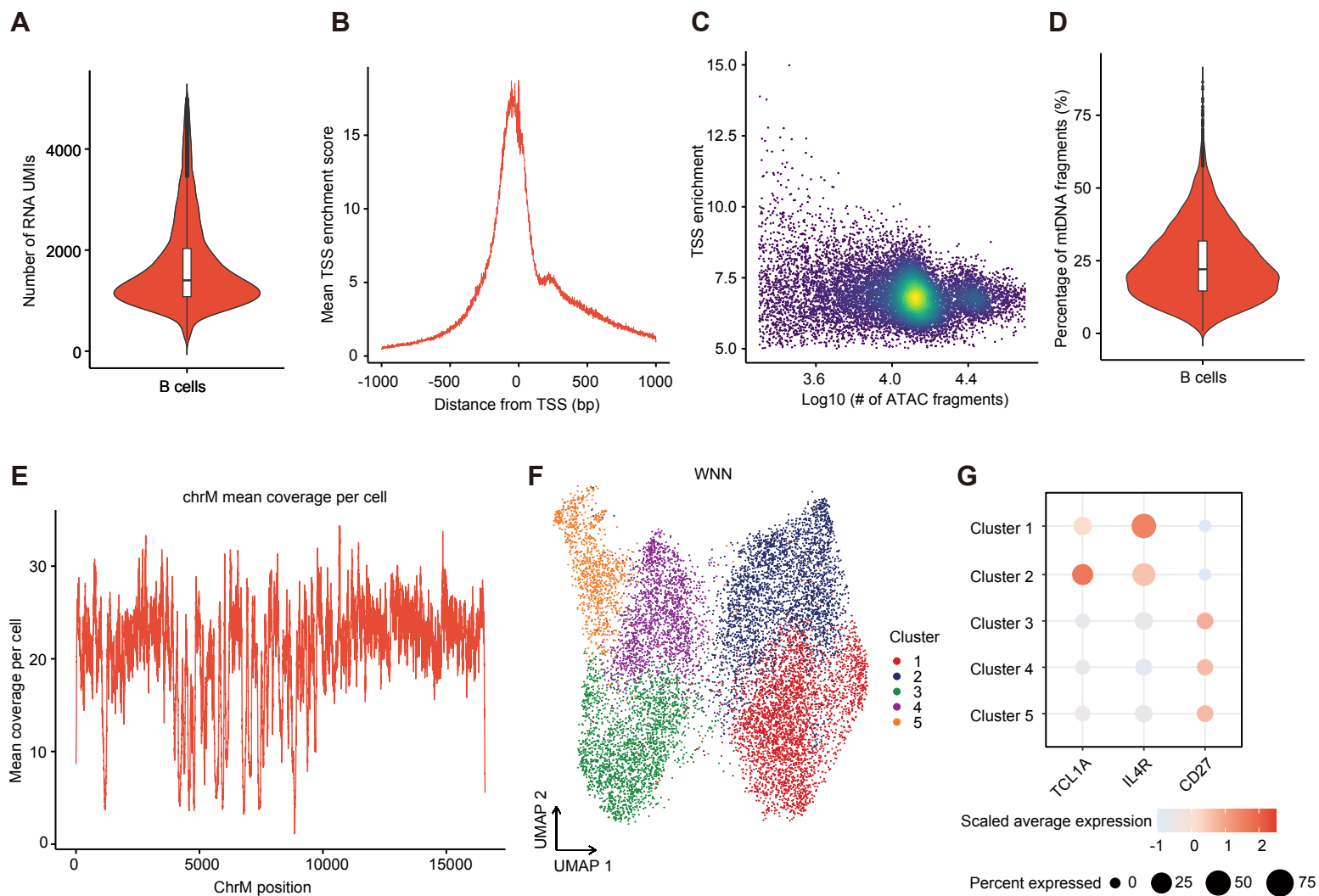

Supplement: qzae073_Supplementary_Data [file qzae073_supplementary_data.zip › Figure S10.pdf]

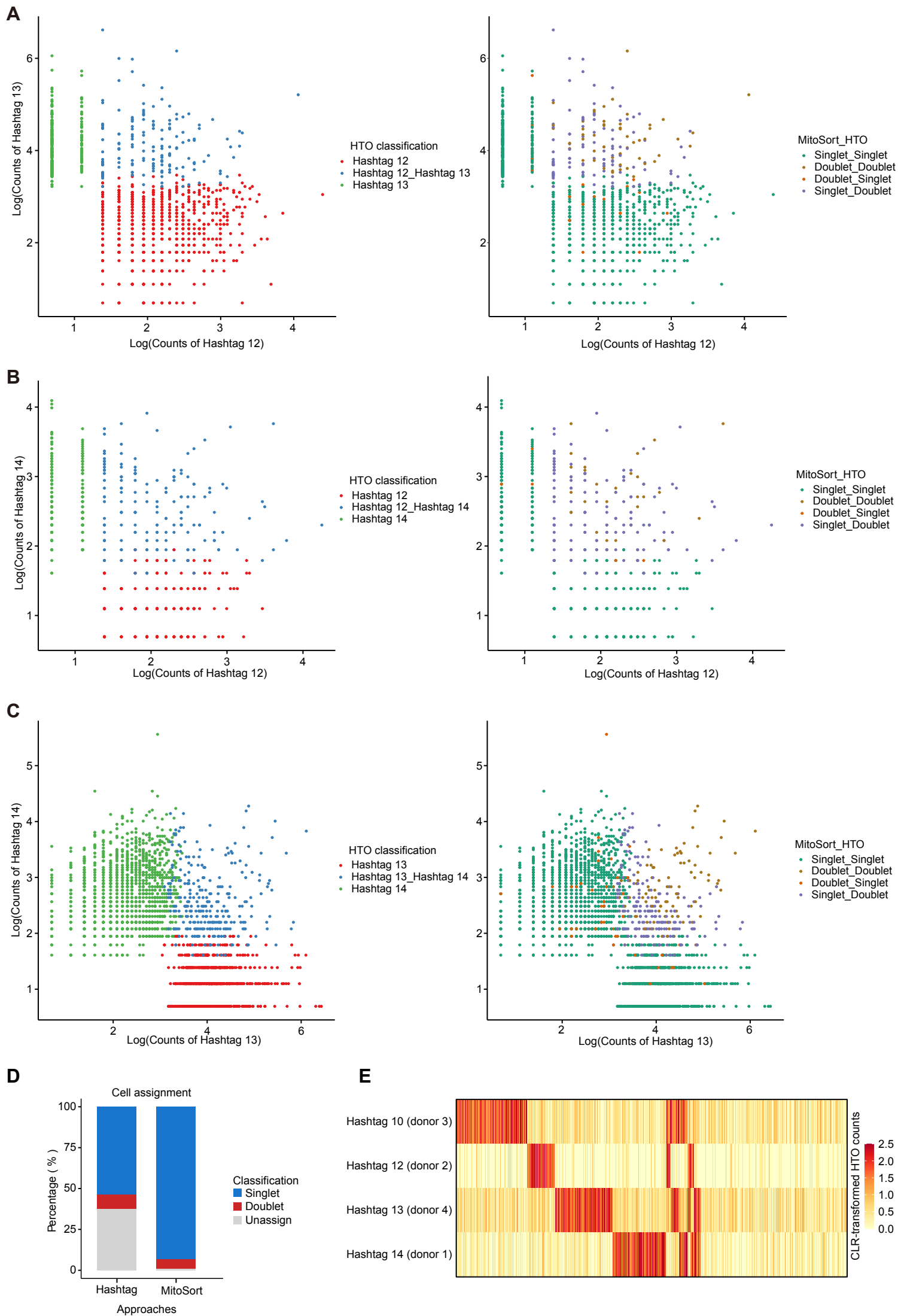

Supplement: qzae073_Supplementary_Data [file qzae073_supplementary_data.zip › Figure S7.pdf]

**A**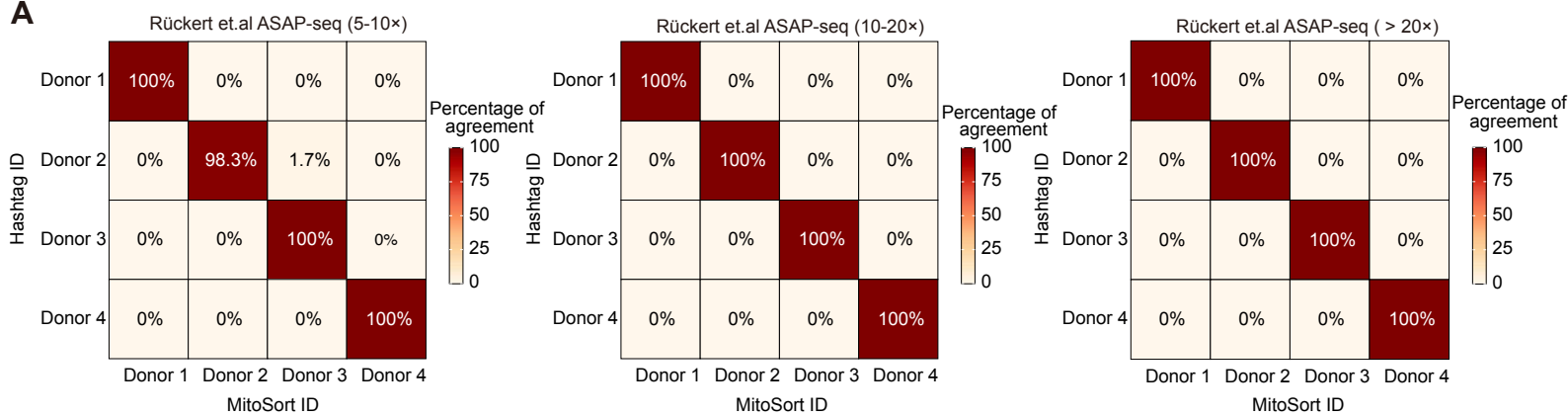**B**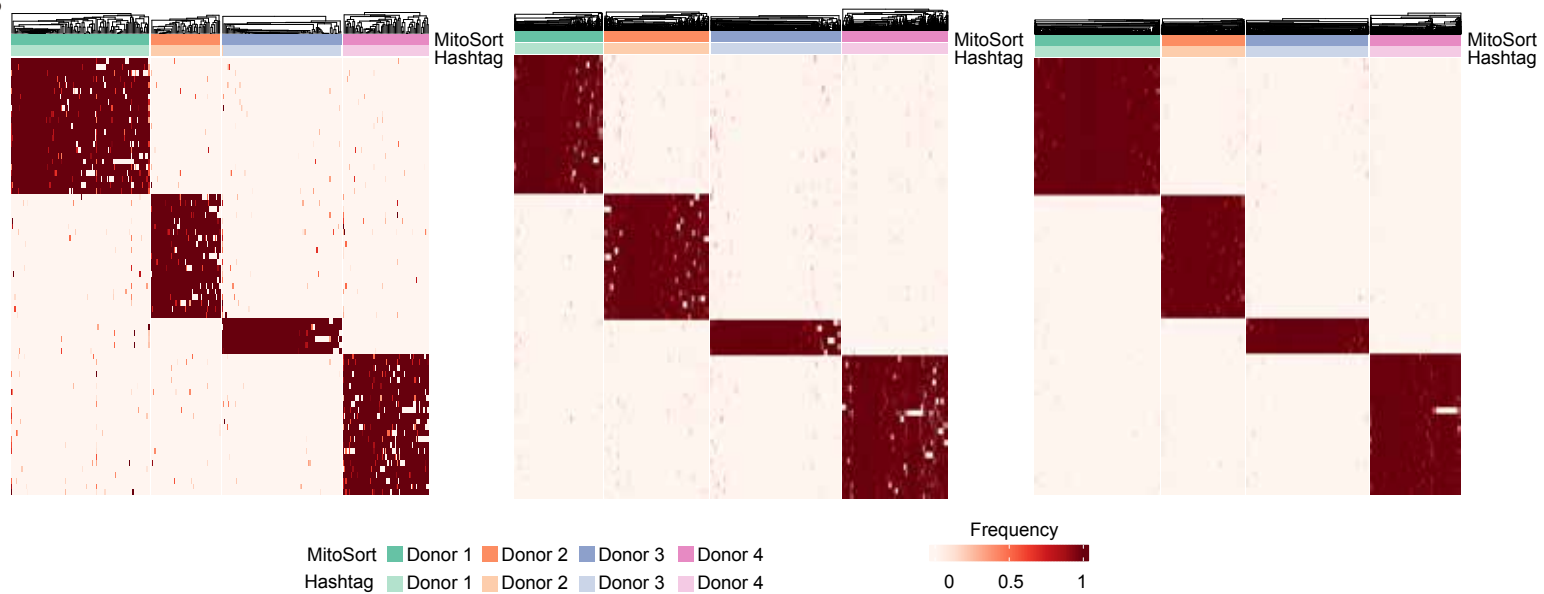

Supplement: qzae073_Supplementary_Data [file qzae073_supplementary_data.zip › Figure S5.pdf]

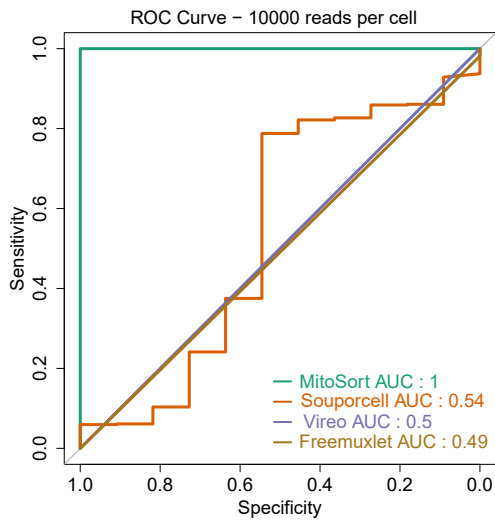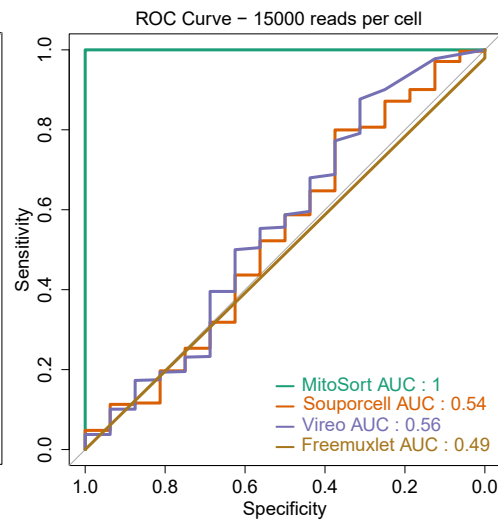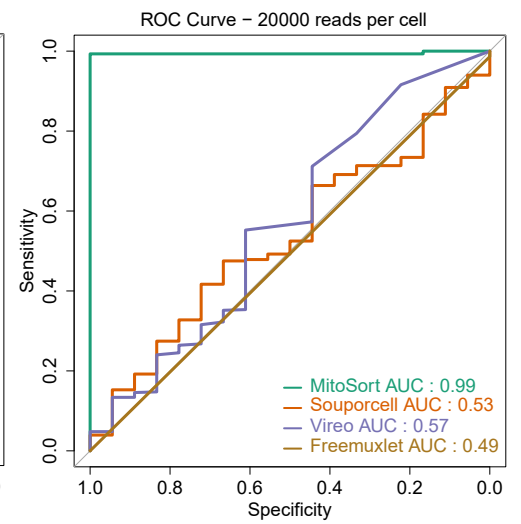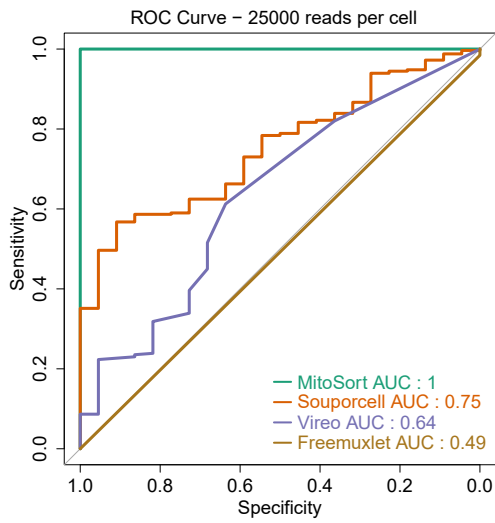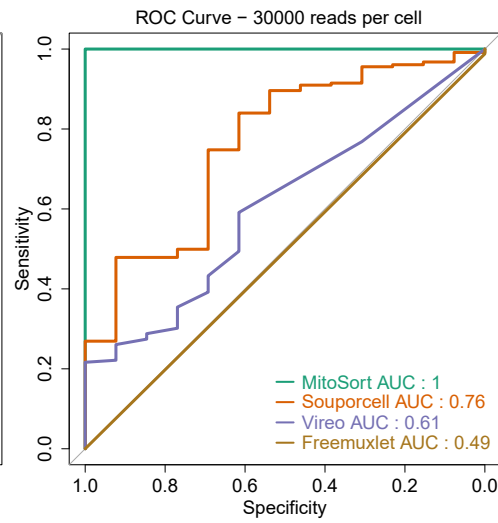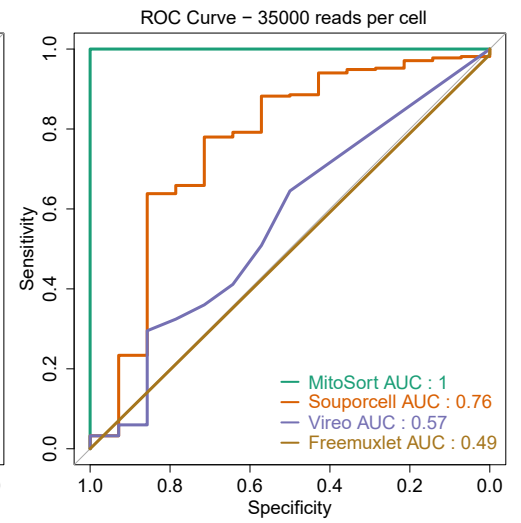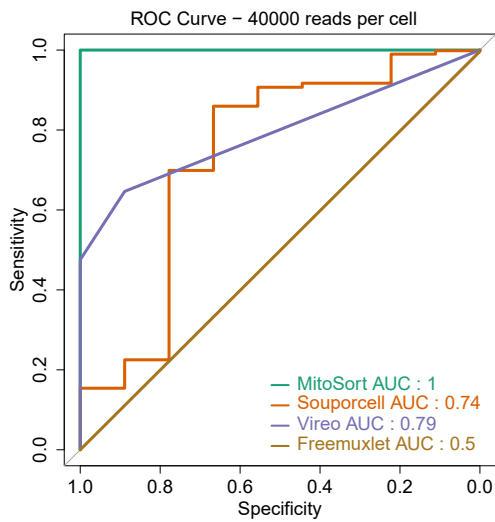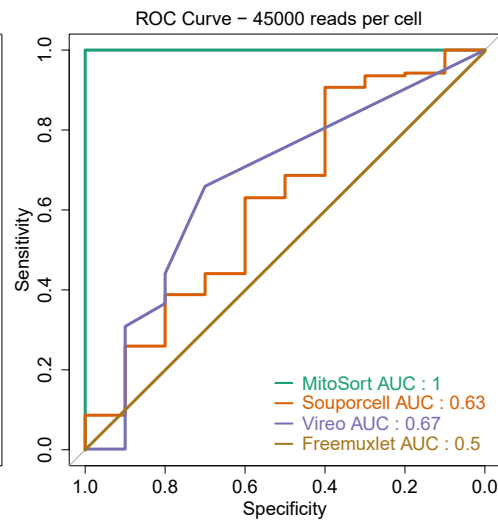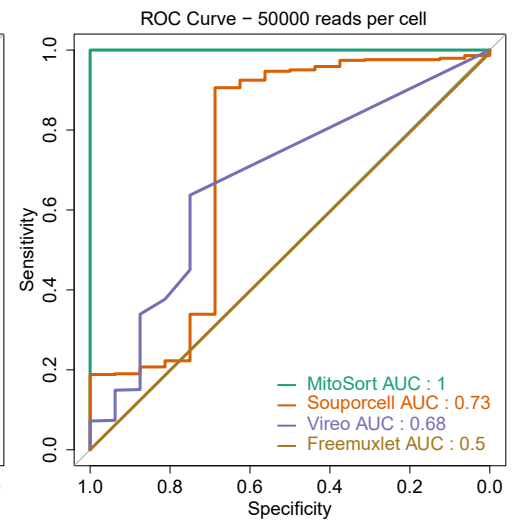

Supplement: qzae073_Supplementary_Data [file qzae073_supplementary_data.zip › Figure S1.pdf]

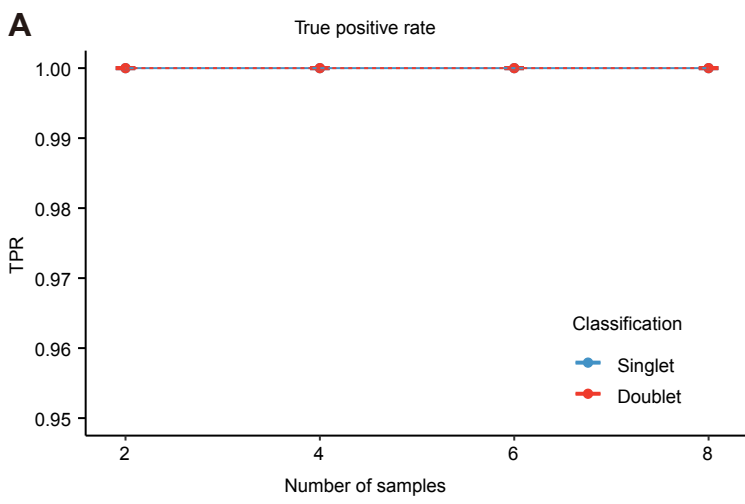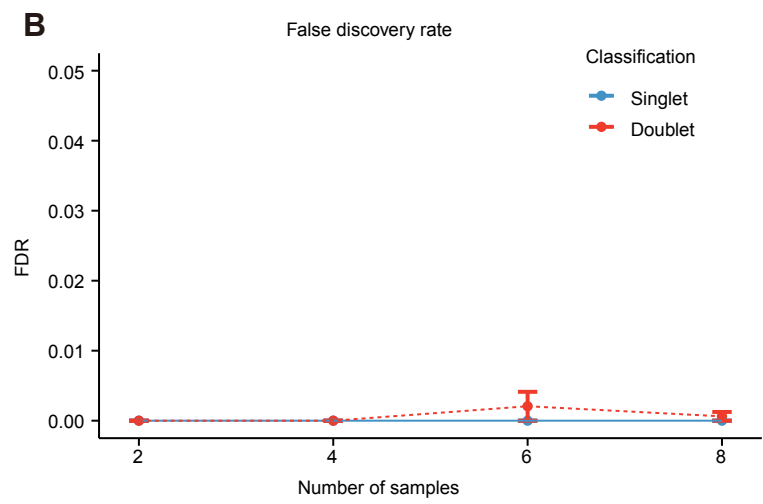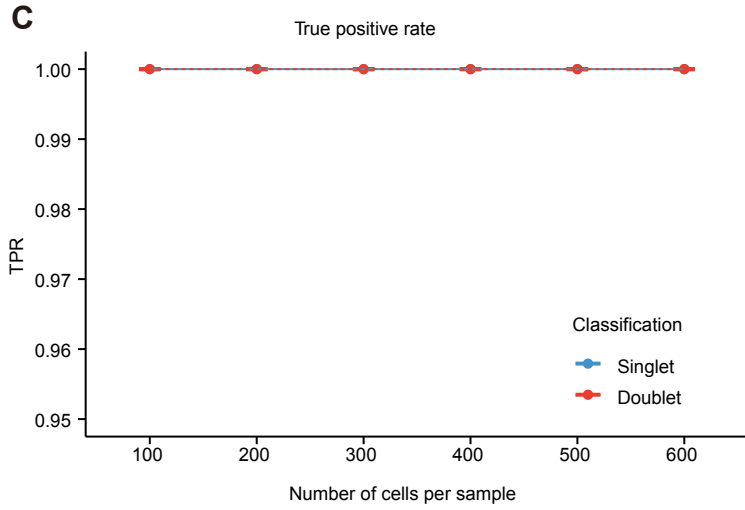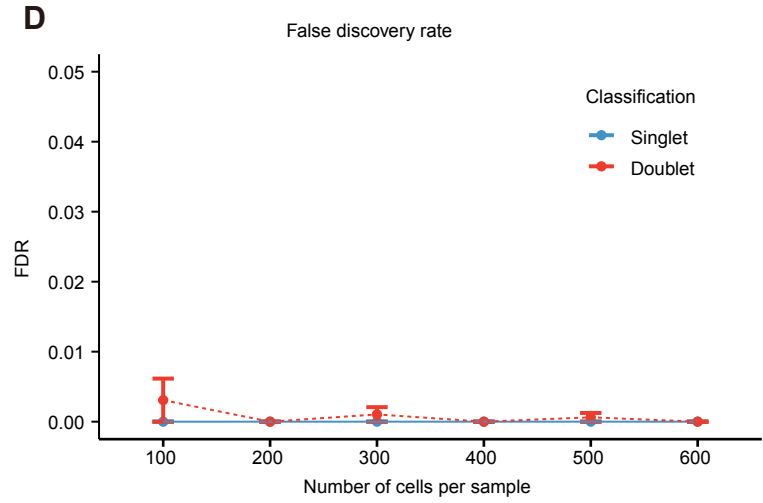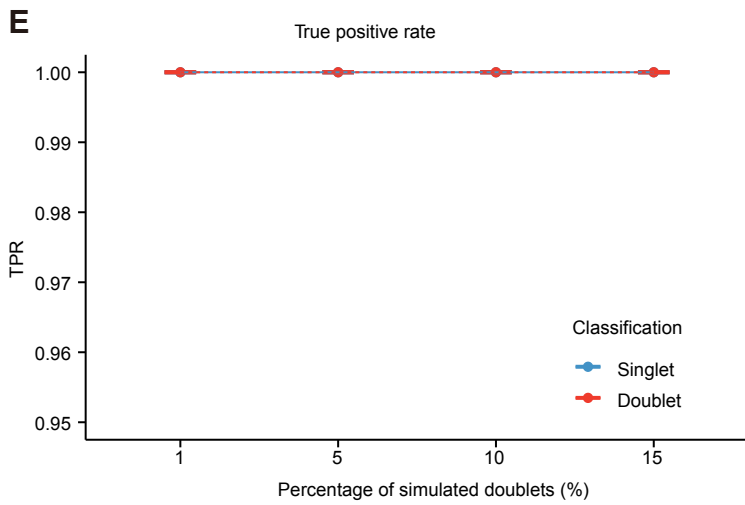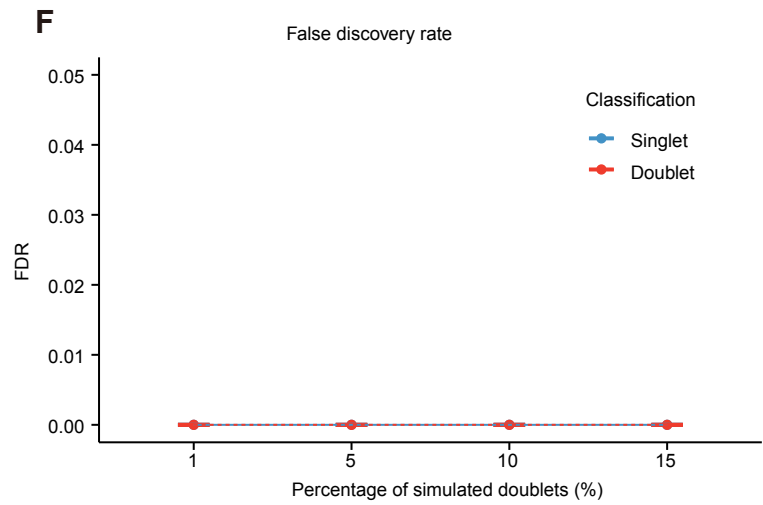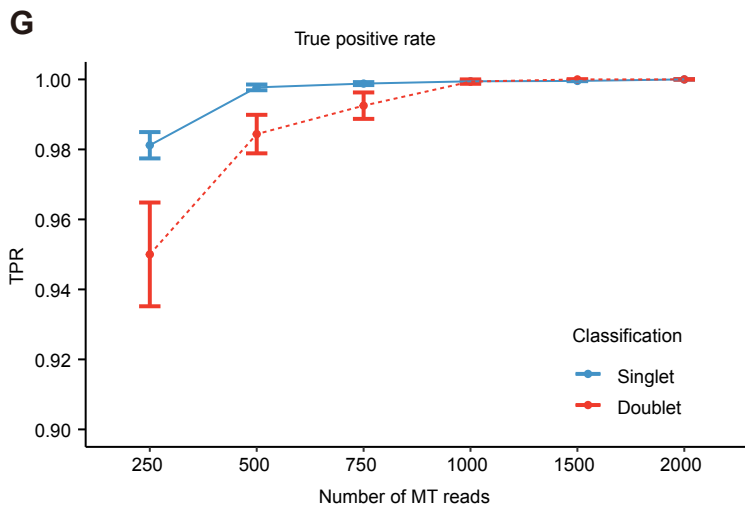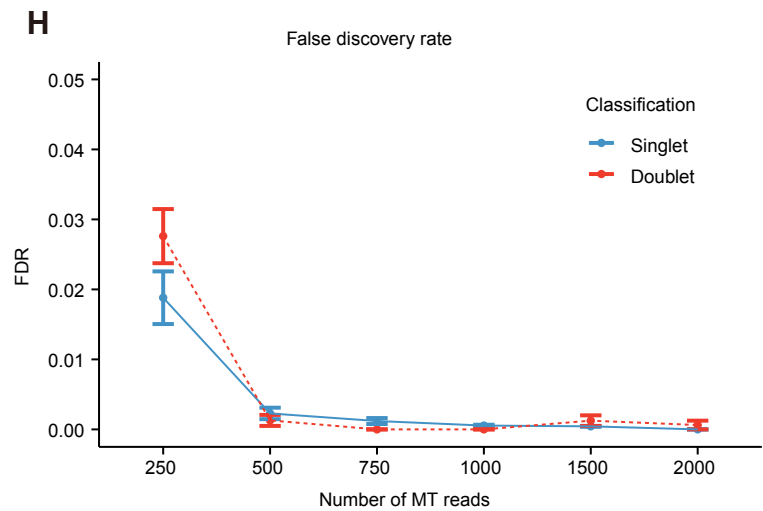

Supplement: qzae073_Supplementary_Data [file qzae073_supplementary_data.zip › Figure S3.pdf]

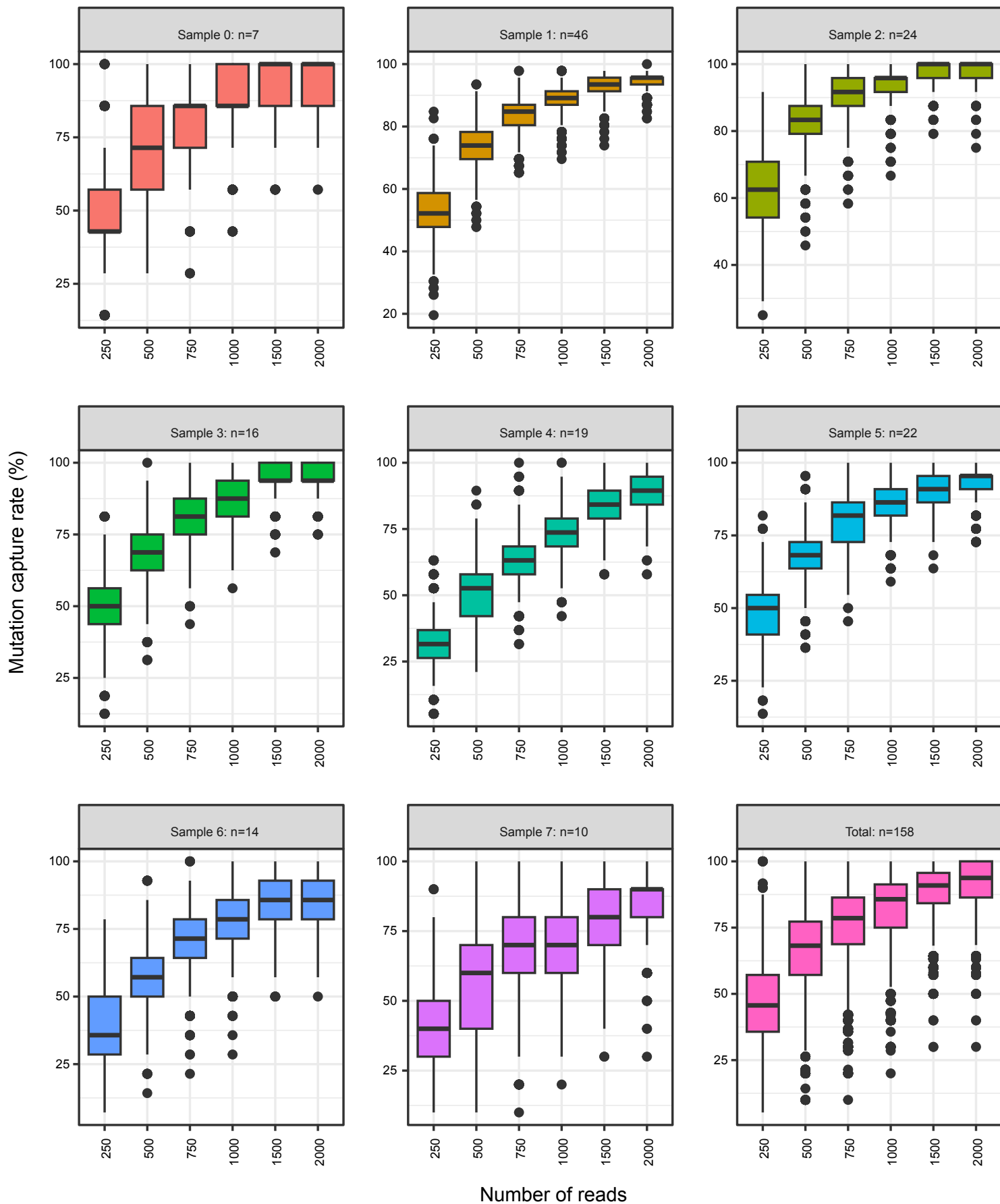

Supplement: qzae073_Supplementary_Data [file qzae073_supplementary_data.zip › Figure S4.pdf]

**A**

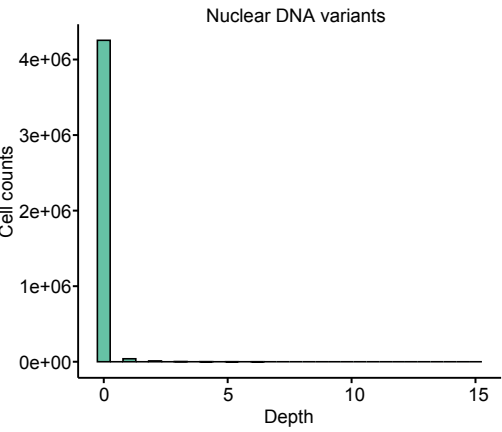

**B**

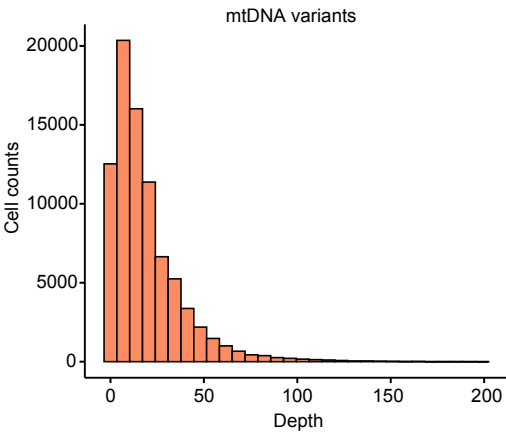

**C**

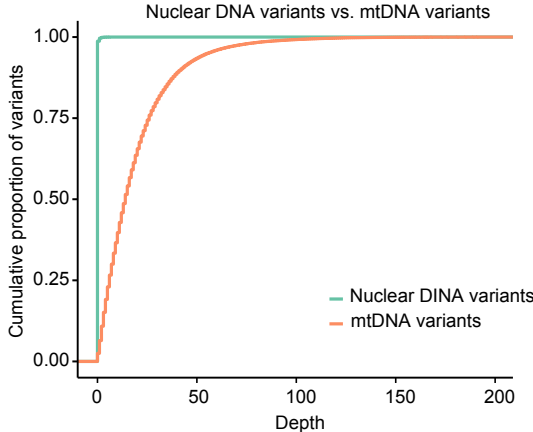

Supplement: qzae073_Supplementary_Data [file qzae073_supplementary_data.zip › Figure S2.pdf]

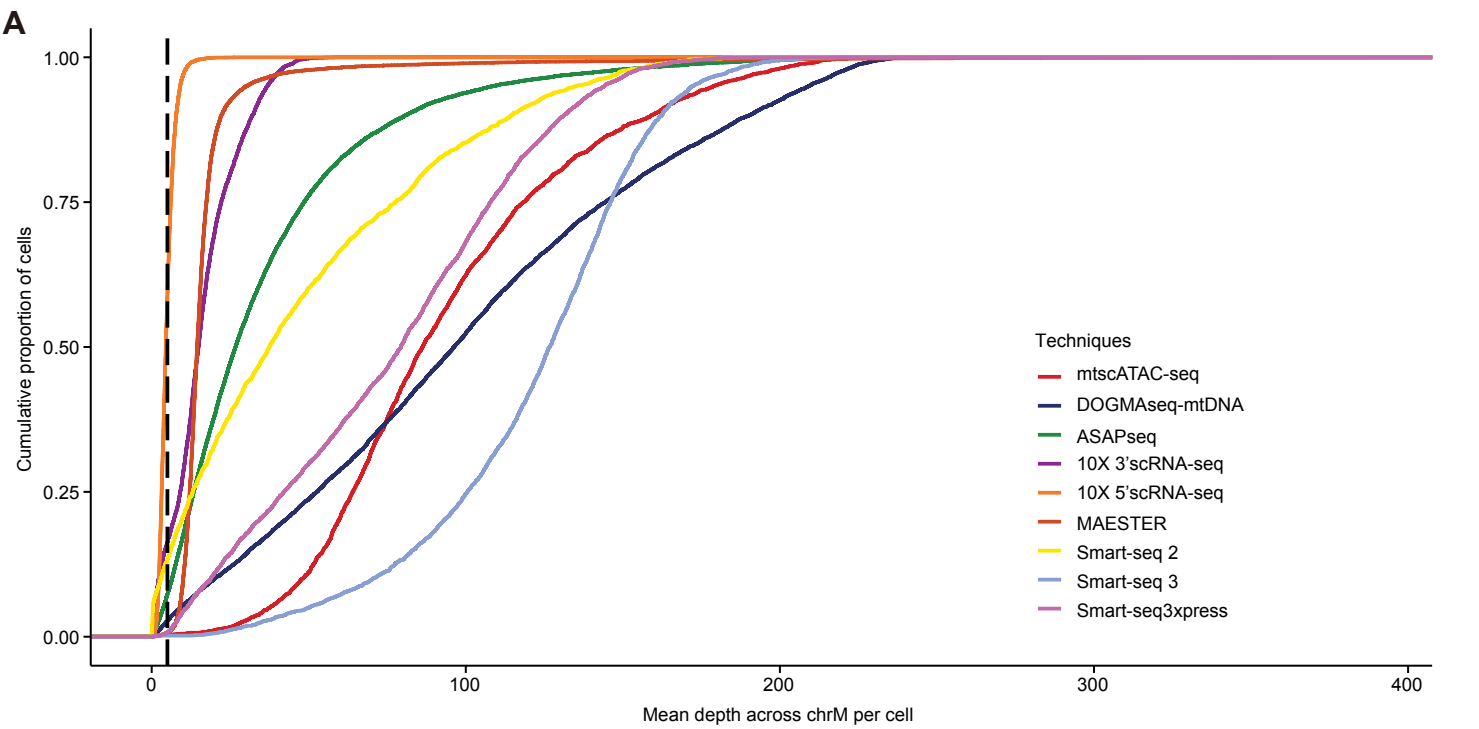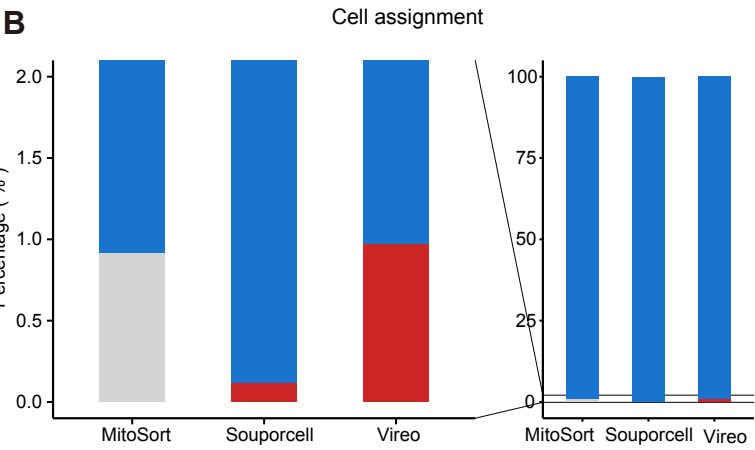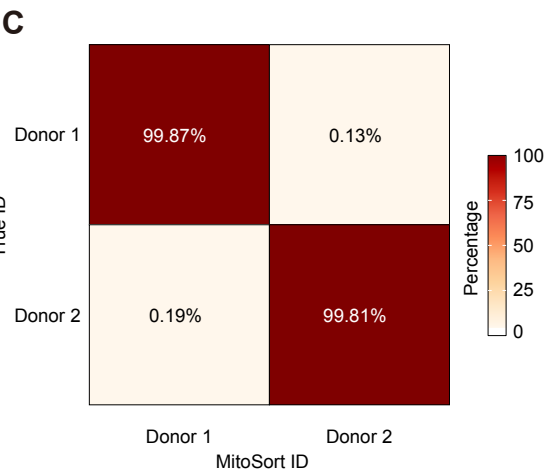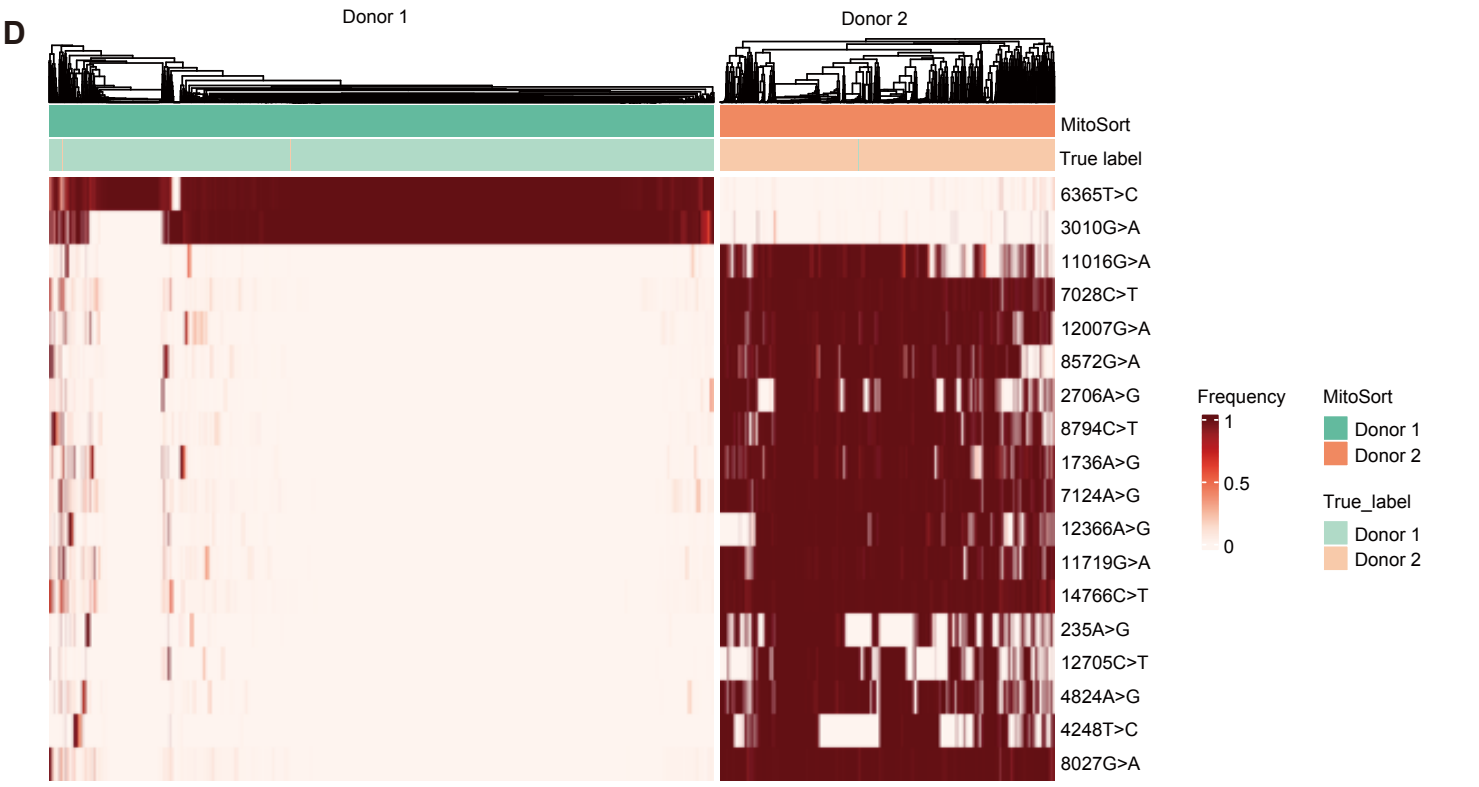

Supplement: qzae073_Supplementary_Data [file qzae073_supplementary_data.zip › Figure S9.pdf]

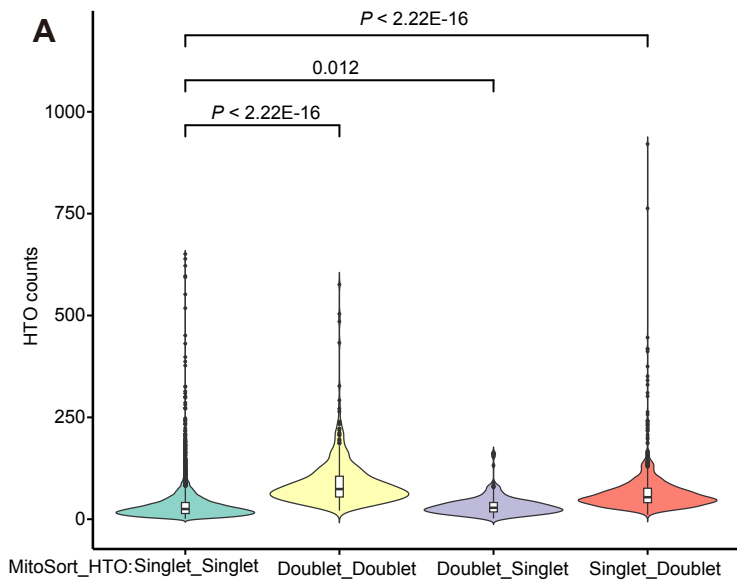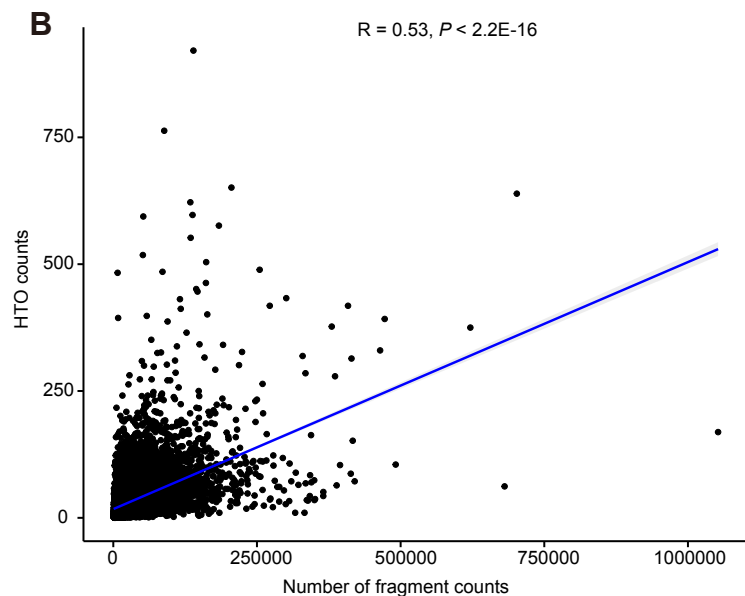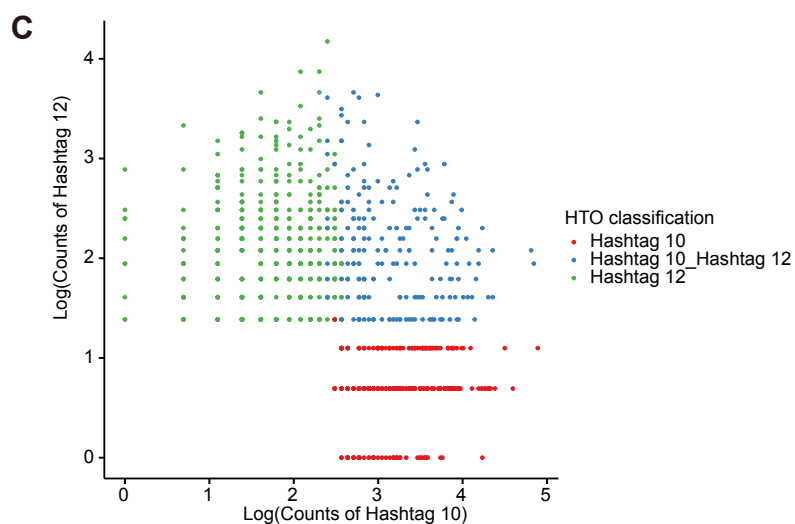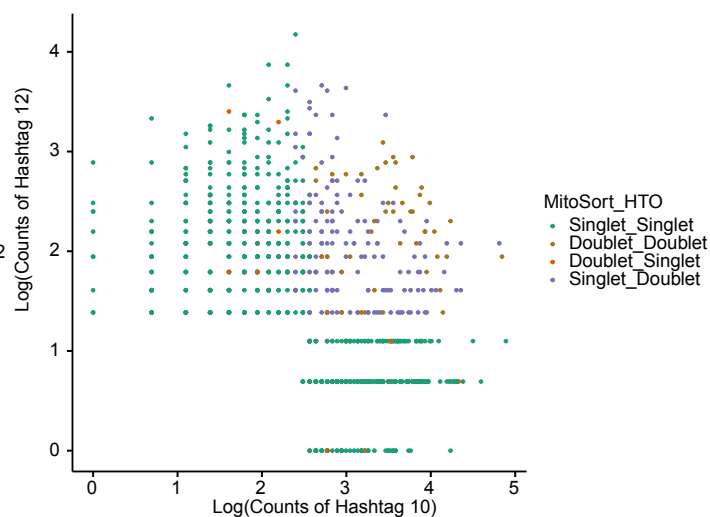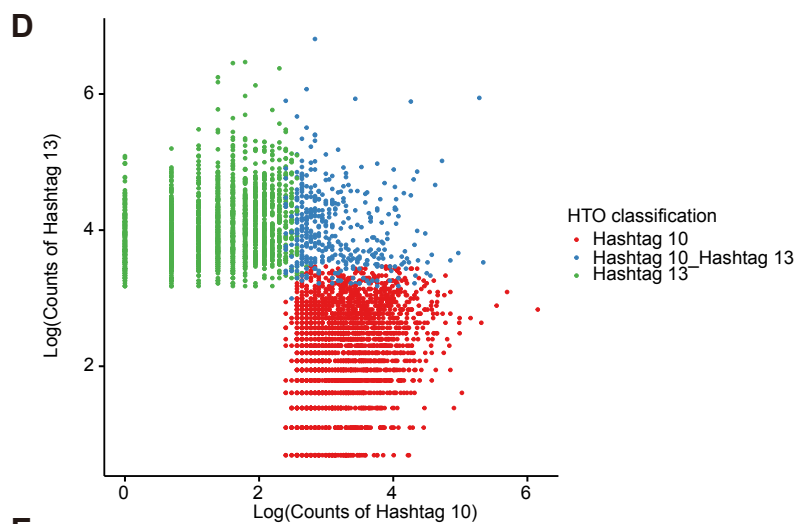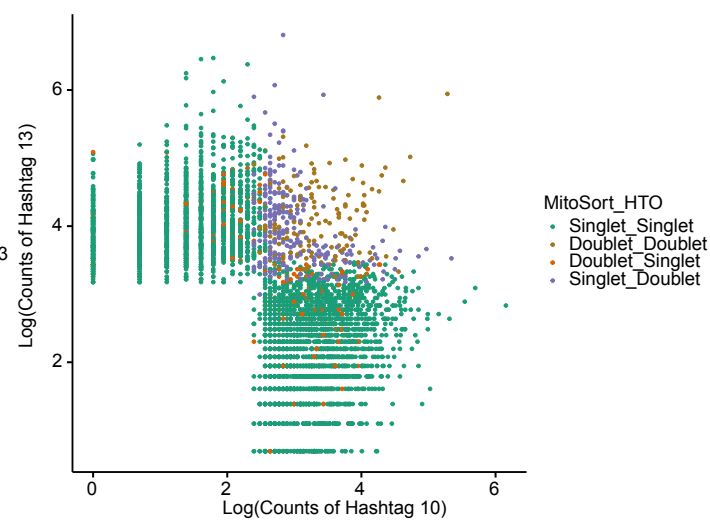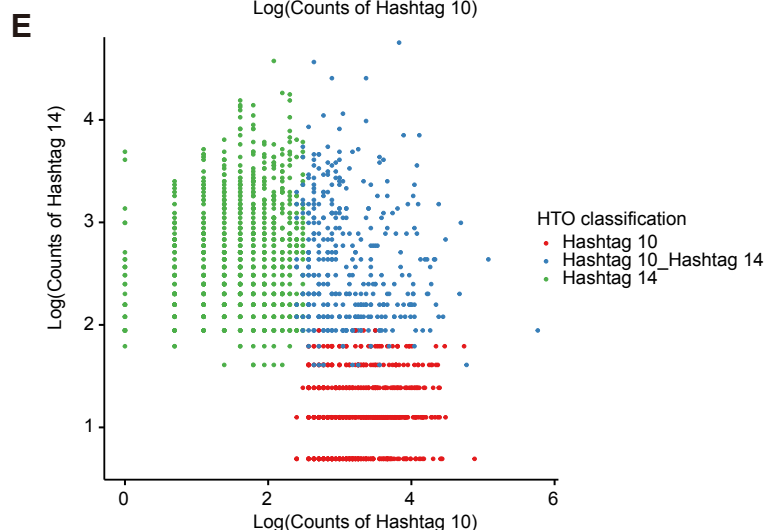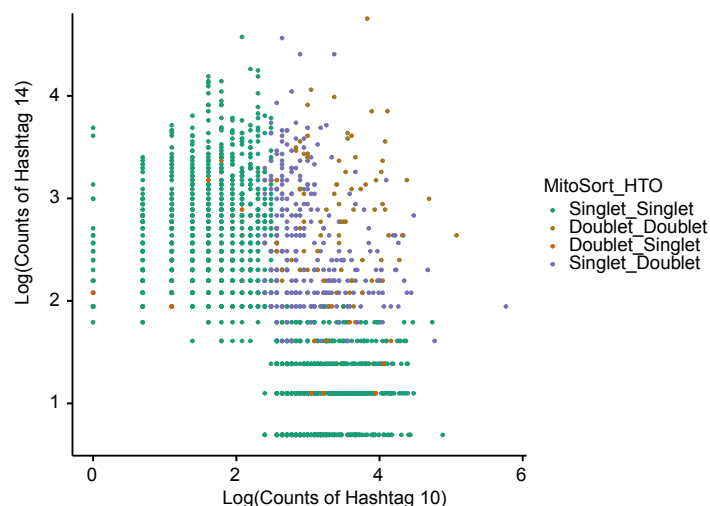

Supplement: qzae073_Supplementary_Data [file qzae073_supplementary_data.zip › Figure S6.pdf]
